# The collagen receptor, discoidin domain receptor 2, functions in Gli1-positive skeletal progenitors and chondrocytes to control bone development

**DOI:** 10.1038/s41413-021-00182-w

**Published:** 2022-02-09

**Authors:** Fatma F. Mohamed, Chunxi Ge, Randy T. Cowling, Daniel Lucas, Shawn A. Hallett, Noriaki Ono, Abdul-Aziz Binrayes, Barry Greenberg, Renny T. Franceschi

**Affiliations:** 1 Department of Periodontics & Oral Medicine, University of Michigan, Ann Arbor, MI USA; 2 Division of Cardiovascular Medicine, University of California at San Diego, San Diego, CA USA; 3 Division of Experimental Hematology and Cancer Biology, Cincinnati Children’s Medical Center, Cincinnati, OH USA; 4 Department of Pediatrics, Cincinnati Children’s Hospital, Cincinnati, OH USA; 5 Department of Orthodontics & Pediatric Dentistry, University of Michigan, Ann Arbor, MI USA; 6 Department of Prosthetic Dental Sciences, College of Dentistry, King Saud University, Riyadh, Saudi Arabia; 7 Department of Biological Chemistry, School of Medicine, University of Michigan, Ann Arbor, MI USA; 8 Department of Biomedical Engineering, University of Michigan, Ann Arbor, MI USA

**Keywords:** Bone, Calcium and phosphate metabolic disorders

## Abstract

Discoidin Domain Receptor 2 (DDR2) is a collagen-activated receptor kinase that, together with integrins, is required for cells to respond to the extracellular matrix. *Ddr2* loss-of-function mutations in humans and mice cause severe defects in skeletal growth and development. However, the cellular functions of *Ddr2* in bone are not understood. Expression and lineage analysis showed selective expression of *Ddr2* at early stages of bone formation in the resting zone and proliferating chondrocytes and periosteum. Consistent with these findings, *Ddr2*^*+*^ cells could differentiate into hypertrophic chondrocytes, osteoblasts, and osteocytes and showed a high degree of colocalization with the skeletal progenitor marker, Gli1. A conditional deletion approach showed a requirement for *Ddr2* in *Gli1*-positive skeletal progenitors and chondrocytes but not mature osteoblasts. Furthermore, *Ddr2* knockout in limb bud chondroprogenitors or purified marrow-derived skeletal progenitors inhibited chondrogenic or osteogenic differentiation, respectively. This work establishes a cell-autonomous function for *Ddr2* in skeletal progenitors and cartilage and emphasizes the critical role of this collagen receptor in bone development.

## Introduction

In addition to its structural function, the collagen-rich extracellular matrix (ECM) of bone has important role in establishing and maintaining cellular organization through control of proliferation, migration, differentiation, and remodeling.^1–3^ These ECM functions are controlled by receptor-mediated interactions with progenitor cells. Disruption of cell-ECM interactions has been implicated in a wide spectrum of human skeletal disorders, including skeletal dysplasias, chondrodysplasias, osteoarthritis, and osteoporosis.^1,4–6^ Specific interactions between collagens and skeletal progenitors are mediated by cell-surface receptors, principally β1 integrins and discoidin domain receptors (DDRs).^7,8^

The principal collagen-binding integrins, α1β1, α2β1, α10β1, and α11β1, are widely expressed in bone cells such as bone marrow precursor cells, chondrocytes, osteoblasts, and osteocytes.^9–12^ β1 integrin disruption in various bone cell lineages results in skeletal and craniofacial phenotypes of varying severity.^13,14^ Mice with β1 integrin knockout in *Twist2*-positive mesenchymal progenitors can still form a mineralized skeleton but die at birth, possibly due to umbilical cord or vascular defects, while disruption in pre-osteoblasts using *Osx-Cre* results in viable mice with moderate early bone defects that become increasingly milder with age. In contrast, knockout in mature osteoblasts had only minor effects on skeletal phenotype.^14^ The observation that mineralized tissue can still form in the absence of integrin function suggests the involvement of other collagen receptors.

DDRs are a second class of collagen receptors having important skeletal functions. Both mammalian members of this family, DDR1 and DDR2, represent an unusual class of receptor tyrosine kinases (RTKs) that are activated by triple-helical collagens.^8,15,16^ Unlike canonical RTKs that bind to soluble ligands, DDRs bind to fibrillar and non-fibrillar collagens with varying specificities and affinities. While both DDR1 and DDR2 bind to type I–III and V fibrillar collagens, only DDR1 binds to the basement membrane type IV collagen and only DDR2 binds to non-fibrillar type X collagen.^15–17^ Structurally, DDRs consist of a discoidin (DS) domain, followed by a DS-like domain, an extracellular juxtamembrane domain, a transmembrane (TM) domain, an intracellular juxtamembrane domain, and the conserved cytoplasmic tyrosine kinase domain. The DS domain, a 160-amino acid motif in the extracellular region, structurally distinguishes DDRs from other RTKs and is required for recognition and binding to fibrillar collagens.^18,19^ DDRs recognize a common motif in fibrillar collagens containing the sequence, GVMGFO, that must be in a triple-helical conformation for efficient binding.^20^ This sequence is distinct from integrin-binding regions of collagen that contain the sequence, GFOGER.^21^ Human and animal genetic studies indicate that DDR2 is an important regulator of bone growth and skeletal development. *DDR2* loss of function mutations in humans causes the rare autosomal recessive growth disorder, spondylo-meta-epiphyseal dysplasia (SMED) with short limbs, and abnormal calcifications (SMED, SL-AC).^22–26^ This disorder is characterized by disproportionate short stature, short limbs, short broad fingers, abnormal metaphyses and epiphyses, platyspondyly and abnormal calcifications.^27,28^ Globally, *Ddr2*-deficient mice share similar phenotypes with SMED, SL-AC patients, including dwarfism, reduced body weight, and skeletal abnormalities characterized by reduced bone mass in axial and appendicular bones, as well as the skull.^29–31^ Changes in bone mass have been attributed to reduced bone formation in the absence of appreciable defects in osteoclast-mediated bone resorption.^30^ However, because global *Ddr2* deficiency also affects other tissues including gonads, heart, and connective tissue, specific cell-autonomous functions of *Ddr2* in the skeleton, if any, cannot be inferred from this work.

Here, we use expression and lineage analysis to identify potential cellular sites of *Ddr2* action and employ a conditional deletion approach to establish critical functions for *Ddr2* in skeletal progenitor cells and chondrocytes to control cartilage and bone formation.

## Results

### *Ddr2* is expressed in long bone growth plates, periosteum, and bone marrow

**Fig. 1:**
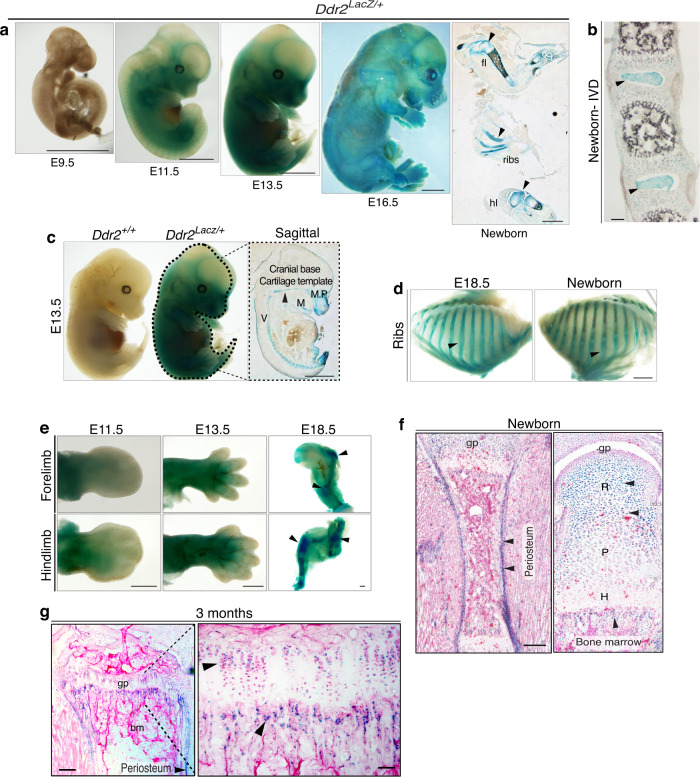
*Ddr2* is highly expressed in long bone growth plates, periosteum and bone marrow. **a** Left, whole-mount X-gal staining of *Ddr2*^*Lacz/+*^ mouse embryos from E9.5-E16.5, showing of *Ddr2* expression starting at E11.5; right, sagittal section (right of midline) of newborn showing forelimb (fl), ribs, and hindlimb (hl) staining. Scale bar: 2 mm. **b** X-gal staining of cryostat section of spine (intervertebral discs) from newborn mice. Scale bar: 100 μm. **c** Side views of *Ddr2*^*+/+*^ (left) *and Ddr2*^*Lacz/+*^ (right) embryos at E13.5. Midline sagittal section (right) of *Ddr2*^*Lacz/+*^ embryo is indicated in dotted lines. Arrowhead points to cranial base cartilage template. M, Meckles cartilage; V, cartilaginous primordia of the vertebral bodies; MP, maxillary process. Scale bar: 2 mm. **d** Side views of rib cages from *Ddr2*^*Lacz/+*^ mice at E18.5 and birth. Scale bar: 2 mm. **e** Whole-mount X-gal staining of *Ddr2*^*Lacz/+*^ embryo forelimbs (upper panel) and hindlimbs (lower panel) at E11.5, E13.5, and E18.5. Scale bar: 500 μm. **f**, **g** X-gal staining of cryostat sections of long bone from newborn and 3 month-old mice. R Resting zone; P Proliferative zone; H Hypertrophic zone; gp Growth plate. Arrowheads indicate areas of positive X-gal staining. Note: Newborn growth plate in (**f**) is a composite of three images. Scale bar: 200 μm (left) and 50 μm (right)

Global *Ddr2* deficiency is associated with severe skeletal defects. However, no information is available concerning the pattern of *Ddr2* expression during bone development. To clarify this issue, we used a *LacZ* “knock-in” allele of *Ddr2* (*Ddr2*^LacZ/+^ mice)^32^ to examine the distribution of *Ddr2* expression from embryonic day E9.5 through 3 months (Fig. 1). As shown in whole mounts of X-gal stained embryos, *Ddr2* expression was first seen at E11.5 (no staining at E9.5) and continued through fetal and postnatal development. More detailed analysis of sagittal sections from E13.5 embryos and newborns showed strong staining in developing skeletal elements in long bones, ribs, vertebrae, cranial base, and maxillary and mandibular processes (Fig. 1a, c, right panels, panel d). LacZ staining was specific and not observed in wild type *Ddr2*^+/+^ embryos (Fig. 1c, left). As shown in whole mounts of developing limbs, staining advanced from proximal regions at E11.5 to distal areas at E13.5 and E18.5 as cartilage and bone formation progressed (Fig. 1e). Cryostat sectioning revealed that this staining was absent from distal mesenchymal condensations of the developing digits but present in more proximal elements specifically in the resting and proliferative zones, but not in the hypertrophic zone, and also in perichondrium (Supplementary Fig. S1). Post-sectioning X-gal staining also detected intense *Ddr2* expression in the growth plate, metaphysis and periosteum in newborn and adult mice (Fig. 1f, g). In growth plates, *Ddr2* showed a gradient of expression; high in resting zone chondrocytes (R), lower in the proliferating zone (P), and lowest in hypertrophic cells (H). In the metaphysis, staining was in the marrow and on trabecular surfaces (Fig. 1g), but conspicuously absent from cortical and trabecular osteocytes (Supplementary Fig. S2). A similar expression pattern was seen in vertebral growth plates (Fig. 1b). *Ddr2* expression was also seen in intervertebral discs with strongest staining in the nucleus pulposus and weaker staining in the annulus fibrosus (Fig. 1b). In summary, initial *Ddr2* expression was coincident with the onset of bone formation with the strongest staining in the growth plate resting zone, metaphysis, perichondrium, and periosteum. In contrast, *Ddr2* expression was low or undetectable in terminally differentiated cells such as hypertrophic chondrocytes and osteocytes.

### *Ddr2*^*mer-iCre-mer*^ marks cells at early stages of bone formation that differentiate to hypertrophic chondrocytes, osteoblasts, and osteocytes

**Fig. 2:**
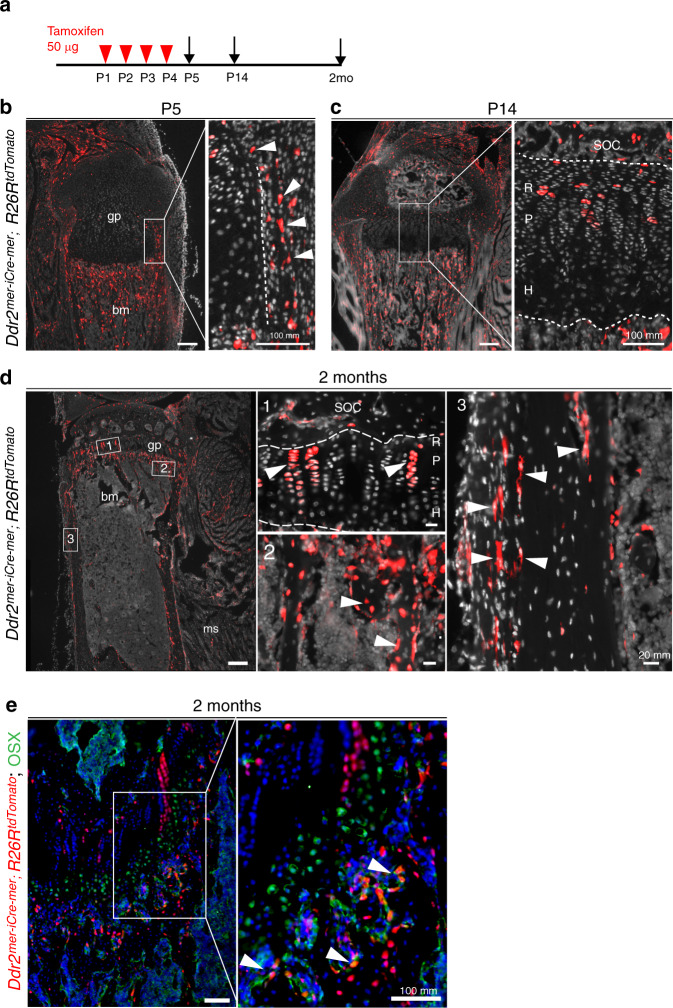
Skeletal contribution of *Ddr2*-expressing cells. **a** Protocol. **b**–**d**, Lineage tracing in *Ddr2*^*mer-iCre-mer*^; *R26R*^*tdTomato*^ mice at the postnatal day 5 (P5), P14 and 2 months (*n* = 2 mice). Red: *Ddr2*-positive cells; gray: cell nuclei; gp Growth plate; bm Bone marrow; SOC Secondary ossification center; ms Muscle; R Resting zone; P Proliferative zone; H Hypertrophic zone. **b** Fluorescent tdTomato (red) distribution in the proximal tibia at P5 showing initial labeling in perichondrium, periosteum, primary spongiosa, and sporadic cells in the growth plate. Scale bar: 200 μm. The boxed region is shown in higher magnification (right). Arrowheads indicate expression in perichondrium. Scale bar: 100 μm. **c** Fluorescent tdTomato distribution at P14 showing label in the resting and proliferative zones of growth plate and in primary spongiosa, but not in the hypertrophic zone. Scale bar: 200 μm. Boxed region is shown in higher magnification (right) Scale bar: 100 μm. Dotted Line denotes chondro-osseous junction. **d** Fluorescent tdTomato distribution at 2 months showing persistence of tdTomato labeling and contribution to (1) growth plate columns, (2) bone cells in primary spongiosa, and (3) periosteum and osteocytes in cortical bone (arrowheads). Scale bar: 200 μm. Images at a higher magnification for different areas of tibia are shown on the right side. Scale bar: 20 μm. **e** High power image of 2-month growth plate and trabecular regions showing co-staining of *tdTomato*^+^ cells with an anti-OSX antibody (green) in osteoblasts on the trabecular surface. Scale bar: 100 μm

The next series of experiments identified the progeny of *Ddr2*-expressing cells by conducting lineage tracing with *Ddr2*^*mer-iCre-mer*^*;R26R-tdTomato* mice (see Methods and Supplementary Fig. S3, S4 for development of *Ddr2*^*mer-iCre-mer*^ mice). *R26R*-tdTomato mice have a flanked STOP cassette that interferes with transcription of tdTomato red fluorescent protein. Upon tamoxifen administration, Cre expression driven by the *Ddr2* promoter excises the LoxP flanked STOP cassette resulting in tdTomato fluorescent protein only in *Ddr2*-expressing cells and their progeny. Cre-mediated recombination was induced with daily intragastric tamoxifen (TAM) injections at P1-P4 (Fig. 2a). Long bones were then harvested at P5, P14, and 2 months and cryosections were analyzed by fluorescence microscopy. No fluorescence was observed in the absence of tamoxifen treatment (result not shown). At P5, analysis of the proximal tibia revealed tdTomato fluorescence in a few cells in the growth plate resting zone and strong fluorescence in the perichondrium, metaphysis, and periosteum (Fig. 2b). This distribution is similar to that seen for *Ddr2-lacZ* staining in Fig. 1; however, in growth plates, the *tdTomato* labeling was considerably more restricted with only a few cells in upper portion showing fluorescence. In bone marrow, labeling was mainly in the metaphysis in association with trabecular bone. After 2 weeks, tdTomato-labeled cells persisted in the periosteum and bone marrow and expanded throughout resting and proliferative zones constituting the uppermost cells in proximal tibial growth plates. However, they were not detected in the hypertrophic region (Fig. 2c inset). After a 2-month chase, tdTomato-labeled cells contributed to chondrogenic and osteogenic lineages (Fig. 2d). Labeled cells expanded to form long columns of stacked cells beginning from the top of the tibial growth plates and extending to the hypertrophic region, suggesting that *Ddr2*-expressing cells can form the entire chondrogenic lineage (Fig. 2d, inset 1). TdTomato labeling was also observed on trabecular, endosteal, and periosteal surfaces and in osteocytes. To confirm that tdTomato fluorescence on trabecular surfaces was in osteoblasts, positive co-staining was demonstrated using an anti-OSX antibody (Fig. 2e). This indicates that *Ddr2*^*mer-iCre-mer*^ labeled cells also contributed to the osteoblast lineage and eventually osteocytes in trabecular and cortical bones.

**Fig. 3:**
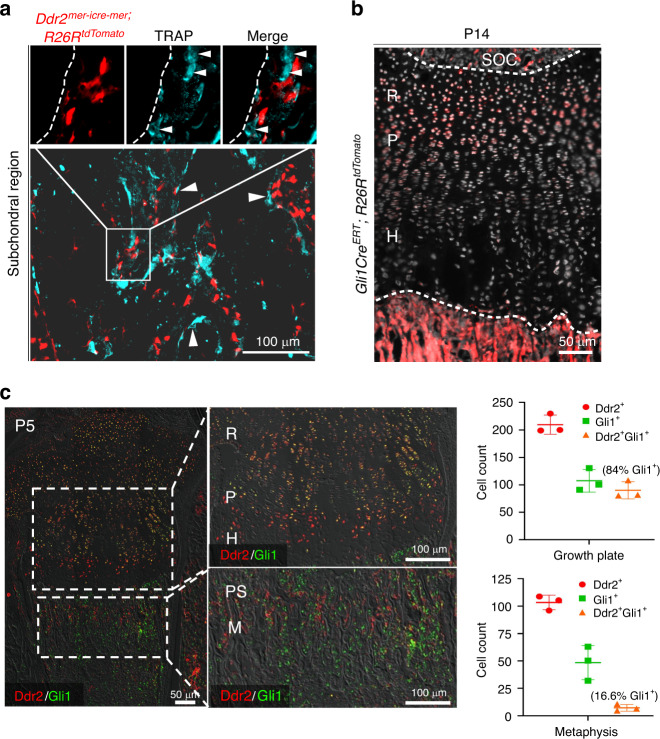
*Ddr2*-derived cells are distinct from osteoclasts; overlapping distribution of *Ddr2* and GLI1. **a** Fluorescence-based TRAP staining on frozen sections from *Ddr2*^*mer-iCre-mer*^; *R26R*^*tdTomato*^ mice at P14 showing no overlap between TRAP^+^ osteoclasts and *Ddr2* tdTomato-labeled cells or their progeny. Scale bar: 50 μm. Arrowheads indicate multinucleated osteoclasts (Cyan). Dotted Line denotes trabecular bone surface. The boxed region is shown in higher magnification (top). **b** *Gli1-Cre*^*ERT*^; *R26R*^*tdTomato*^ mice were treated with tamoxifen as in Fig. 2a and examined at P14. Section of the proximal tibia showing *Gli1-Cre*^*ERT*^ fluorescent tdTomato fluorescence with intense labeling in the resting zone of the growth plate and primary spongiosa, in cell populations having partial overlap with *Ddr2*^*mer-iCre-mer*^ *Ddr2-*positive cells in Fig. 2. Scale bar: 100 μm. **c** IF colocalization of Ddr2 (red) and Gli1 (green). Proximal tibias were isolated from P5 C57BL6 mice. Extensive Gli1 and Ddr2 colocalization were seen in the resting zone (R) and proliferative (P) chondrocytes with low staining in hypertrophic cells (H). More limited staining was seen in select cells in primary spongiosa (PS) and metaphysis (M). Right panels, Quantitation of Ddr2^+^, Gli1^+^, and Gli1^+^ Ddr2^+^ cells in growth plate and PS/MS. The percentage of Gli1^+^ cells also containing Ddr2 is shown in brackets. Values are means ± SD with *n* = 3 mice

Osteoclasts are multinucleated giant cells of the hematopoietic lineage involved in bone resorption during the development and remodeling of the skeleton and during tooth eruption.^33^ On the basis of cell culture studies, it was previously proposed that *Ddr2* is expressed in osteoclasts as well as osteoblasts/osteoprogenitors.^34,35^ We performed fluorescent-based TRAP staining to mark TRAP^+^ multinucleated cells on cryostat sections from *Ddr2*^*mer-iCre-mer*^; *R26R*t*dTomato* mice. Cre-recombination was induced at birth with four TAM injections, as described in Fig. 2a, and multiple skeletal tissues were examined after 2 weeks. In a long bone, multinucleated TRAP^+^ cells (cyan) were detected in the subchondral region; however, the spatial distribution of these cells was distinct from that of *Ddr2* tdTomato-labeled cells (red), indicating that there is no overlap between TRAP^+^ osteoclasts and *Ddr2* tdTomato-labeled cells or their progeny (Fig. 3a). Based on these results, *Ddr2*-expressing cells can contribute to chondrogenic and osteogenic lineages but do not form osteoclasts.

Intriguingly, the distribution of *Ddr2* in our experiments was reminiscent of that previously reported for *Gli1*, a mediator of hedgehog signaling and skeletal progenitor cell (SPC) marker. In lineage-tracing experiments, *Gli1-*positive cells were associated with stem cell populations in cranial sutures, resting and proliferative zone chondrocytes and mesenchymal metaphyseal osteoprogenitors in long bones.^36,37^ To examine the possible overlap between *Ddr2* and *Gli1-*positive cells, we used lineage analysis to verify the activity of inducible *Gli1-Cre*^*ERT*^ under our experimental conditions by breeding with *R26R*-tdTomato reporter mice. Newborn *Gli1-Cre*^*ERT*^;*R26R*-tdTomato mice were injected with TAM using the same conditions used for *Ddr2*^*mer-iCre-mer*^;*R26R*-tdTomato mice and analyzed for tdTomato distribution at P14. Similar to previous results,^37^ tdTomato labeling was detected in periosteum and metaphysis of long bones as well as cranial sutures (not shown). The label was also detected in long bone growth plates, including in most cells in the resting zone and early proliferative zone (Fig. 3b). This pattern is like that seen with *Ddr2* labeling, suggesting partial overlap between *Ddr2*- and *Gli1*- expressing cells and their progeny. More direct evidence for colocalization of Ddr2 and Gli1 is shown in Fig. 3c using double immunofluorescence labeling of the proximal tibia from P5 mice. Consistent with the *Ddr2-LacZ* distribution shown in Fig. 1, Ddr2-specific staining (red) was detected in resting and proliferative zones of the growth plate and some marrow cells in the metaphysis. Gli1 (green) showed considerable overlap with Ddr2 particularly in growth plates. Quantitation of Ddr2-Gli1 colocalization is shown in Fig. 3c. Approximately 84 percent of Gli1^+^ cells in growth plate cells were Ddr2^+^ while only 17 percent of Gli1^+^ cells in the metaphysis also contained Ddr2.

### Requirement for *Ddr2* in specific bone cell populations

From the above results, we conclude that *Ddr2* is preferentially localized to the skeleton with initial expression coincident with the onset of bone formation. *Ddr2* is expressed at early stages of bone formation in resting and proliferative zone chondrocytes, bone marrow, and periosteum and low or absent from terminally differentiated cells (e.g., hypertrophic chondrocytes and osteocytes). This distribution at least partially overlapped with the SPC marker, Gli1. Furthermore, lineage tracing with *Ddr2*^*mer-iCre-mer*^; *R26R*-tdTomato mice showed that the progeny of *Ddr2*-positive cells become hypertrophic chondrocytes, osteoblasts and osteocytes, again consistent with *Ddr2* being expressed in the early stages of the bone lineage. However, Ddr2 was also present in metaphyseal osteoprogenitors/osteoblasts on trabecular surfaces, which suggests functions in more mature cells. To assess *Ddr2* functions in specific cell populations, we employed a conditional deletion approach. This was necessary both to determine if *Ddr2* has cell-autonomous functions in the bone as well as to define the specific cell populations involved. As noted in the Introduction, previous studies with globally *Ddr2*-deficient mice (*Ddr2*^*slie/slie*^) established the importance of *Ddr2* in bone formation.^30,31^ However, it was not clear from this work whether actions of *Ddr2* on the skeleton were direct or indirect. Specifically, *Ddr2*^*slie/slie*^ mice have several non-skeletal phenotypes, including gonadal insufficiency and accompanying reductions in levels of circulating sex steroids that could systemically impact bone formation.^31^ This further emphasizes the importance of using a conditional deletion approach to determine if *Ddr2* has cell-autonomous functions. To accomplish this, previously developed *Ddr2*^*fl/fl*^ mice^32^ were crossed with a series of *Cre* mouse lines to selectively delete *Ddr2* in *Gli1*-positive SPCs, chondrocytes and mature osteoblasts/osteocytes.

### Deletion of *Ddr2* in *Gli1*- or *Col2a1*-positive cells inhibits endochondral bone formation

**Fig. 4:**
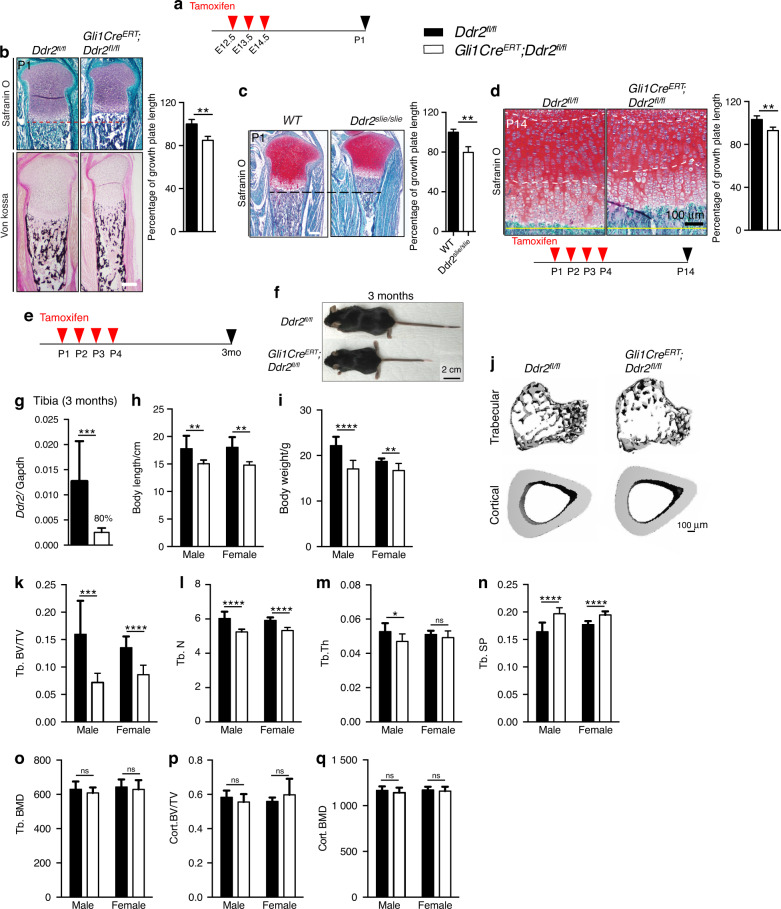
Requirement for *Ddr2* in *Gli1*-positive chondro-osteoprogenitors. **a** Experimental protocol for embryonic study (**b**). **b** Safranin O and von Kossa staining of tibia from *Ddr2*^*fl/fl*^ (*n* = 3) and *Gli1-Cre*^*ERT*^*;Ddr2*^*fl/fl*^ (*n* = 4) newborns. von Kossa staining (lower panel) shows no evident difference in mineralization (black staining). Scale bar: 200 μm. Right; quantification of growth plate length. **c** Safranin O staining of proximal tibia growth plates from WT and *Ddr2*^*Slie/Slie*^ newborns. The dotted line indicates the growth plate lengths of mutant in relation to WT. Scale bar: 200 μm. Right; quantification of growth plate length (*n* = 3). **d** Safranin O staining and quantification (right) showing reduced growth plate lengths in mutant mice at 2 weeks of age (*n* = 3), experimental protocol at the bottom. Scale bar: 100 μm. **e** Experimental protocol for adult study. **f** Gross phenotype of 3 month-old *Ddr2*^*fl/fl*^ and *Gli1-Cre*^*ERT*^*;Ddr2*^*fl/fl*^ littermates. *Gli1-Cre*^*ERT*^*;Ddr2*^*fl/fl*^ mice have skeletal dwarfism (*n* = 20, 10 mice/sex). Scale bar, 2 cm. **g** Gene expression analysis of *Ddr2* from whole 3 month-old tibiae to confirm knockout efficiency (*n* = 6 mice). **h**, **i** Body length (cm) and weight (g) measurements (*n* = 20, 10 mice/sex). **j** 3D micro-computed tomography (microCT) models of the tibial metaphysis and cortical mid-shaft in 3 month-old *Ddr2*^*fl/fl*^ and *Gli1-Cre*^*ERT*^*;Ddr2*^*fl/fl*^ mice. Scale bar 100 µm. **k**–**o** Quantification of trabecular bone volume fraction (Trab. BV/TV), trabecular number (Tb.N), trabecular thickness (Tb.Th), trabecular spacing (Tb.Sp), and bone mineral density (Trab. BMD). (*n* = 20, 10 mice/sex). **p**, **q** Quantification of cortical bone volume fraction (Cort. BV/TV), and bone mineral density (Cort. BMD). Statistics: Data are expressed as mean ± SD. Unpaired t-test, *P < 0.05, **P < 0.01; ***P < 0.001; ****P < 0.000 1; ns, not significant

*Gli1-Cre*^*ERT*^; *Ddr2*^*fl/fl*^ mice were generated and treated with TAM during embryonic development or immediately after birth. In the first case, pregnant dams were given three daily intragastric TAM injections beginning at E12.5 and pups were analyzed at birth (Fig. 4a, b). In the second group, neonates were dosed with TAM at P1-P4 and analyzed after either 2 weeks (Fig. 4d) or 3 months (Fig. 4e–q). *Ddr2* knockout during fetal development led to an approximately 15% reduction in growth plate length at birth compared to *Ddr2*^*f/f*^ controls (Fig. 4b). This was comparable to the reduction seen in globally *Ddr2*-deficient newborns (*Ddr2*^*slie/slie*^ mice, Fig. 4c). However, there were no obvious changes in bone mineralization (von Kossa-positive area, Fig. 4b, lower panel). Similarly, *Gli1-Cre*^*ERT*^-mediated *Ddr2* knockout in neonates that were analyzed at 2 weeks resulted in an approximately 12% reduction in growth plate length (Fig. 4d). A detailed analysis of the skeletal phenotype of 3 month-old mice allowed us to assess the effects of neonatal *Ddr2* inactivation on the young adult skeleton. PCR analysis of genomic DNA extracted from cartilage-containing ear punches was used to demonstrate efficient recombination of the floxed allele in *Gli1-Cre*^*ERT*^; *Ddr2*^*fl/fl*^ mice after TAM injections (Supplementary Fig. S5). Consistent with this result, qRT-PCR analysis of *Ddr2* mRNA extracted from whole tibiae showed an 80% reduction in mutant mice, compared with *Ddr2*^*fl/fl*^ controls (Fig. 4g). *Ddr2* loss in *Gli1*-expressing cells caused a skeletal dwarfism of similar magnitude to global *Ddr2* knockouts (Fig. 4f, h, i). *Gli1-Cre*^*ERT*^;*Ddr2*^*fl/f*^ mice exhibited a significant reduction in the body length both in males and females and a decrease in body weights. The effect of *Ddr2* knockout on trabecular and cortical bone was assessed using μCT. Mutant mice displayed a significant reduction in trabecular bone volume (BV/TV) associated with a decrease in trabecular number and thickness and increase in trabecular spacing, compared with *Ddr2*^*fl/fl*^ controls (Fig. 4j–n). However, no differences in trabecular or cortical bone mineral density (BMD) were observed, nor was cortical BV/TV reduced (Fig. 4o–q). This phenotype is similar to that seen in *Ddr2*^*slie/slie*^ mice.^30^ These results indicate that *Ddr2* functions in *Gli1*-positive SPCs present during development (E12.5) and in neonates. The consequences of *Ddr2* deficiency were first manifested as defects in growth plate-mediated longitudinal growth, but by 3 months also led to defective trabecular, but not cortical bone mass.

**Fig. 5:**
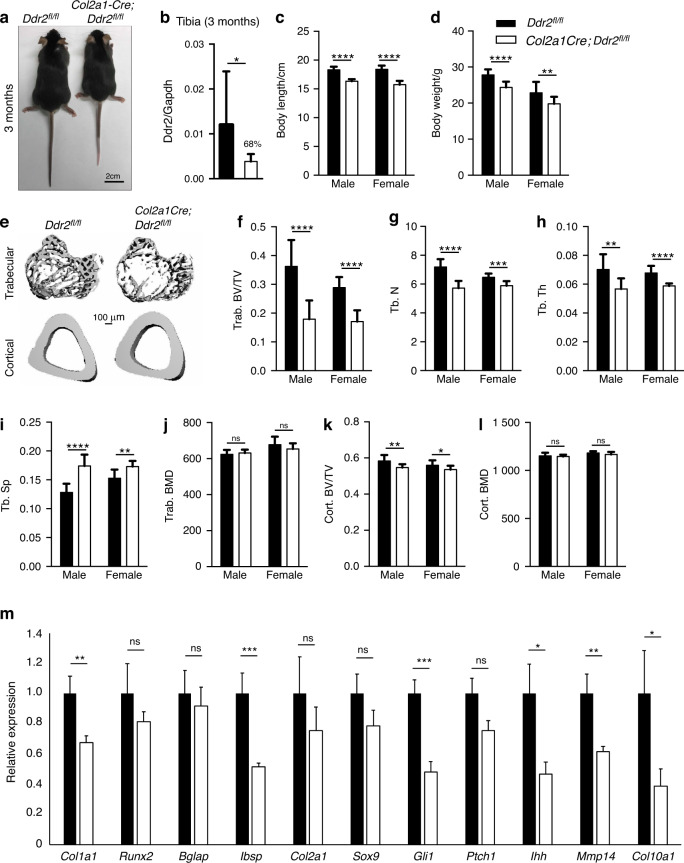
Requirement for *Ddr2* in *Col2a1*-positive chondroprogenitor cells. **a** Gross phenotype of 3 month-old *Ddr2*^*fl/fl*^ and *Col2a1-Cre;Ddr2*^*fl/fl*^ littermates. *Col2a1-Cre; Ddr2*^*fl/fl*^ mice have skeletal dwarfism (*n* = 20, 10 mice/sex). Scale bar: 2 cm. **b** Gene expression analysis of *Ddr2* from whole 3 month-old tibiae to confirm knockout efficiency (*n* = 6 mice). **c**, **d** Measurements of body length (cm) and weight (g) (*n* = 20, 10 mice/sex). **e** 3D micro-computed tomography (microCT) models of the tibial metaphysis and cortical mid-shaft in 3 month-old *Ddr2*^*fl/fl*^ and *Col2a1-Cre; Ddr2*^*fl/fl*^ mice. Scale bar: 100 µm. **f**–**j** Quantitative data of trabecular bone volume fraction (Trab. BV/TV), trabecular number (Tb.N), trabecular thickness (Tb.Th), trabecular spacing (Tb.Sp) and bone mineral density (Trab. BMD), (*n* = 20, 10 mice/sex). **k**, **l** Quantification of cortical bone volume fraction (Cort. BV/TV), and bone mineral density (Cort. BMD), (*n* = 20, 10 mice/sex). **m** Gene expression analysis of osteogenic and chondrogenic differentiation markers. Statistics: Unpaired t-test *P < 0.05, **P < 0.01; ***P < 0.001; ****P < 0.000 1; ns Not significant. Data were presented as mean ± SD

**Fig. 6:**
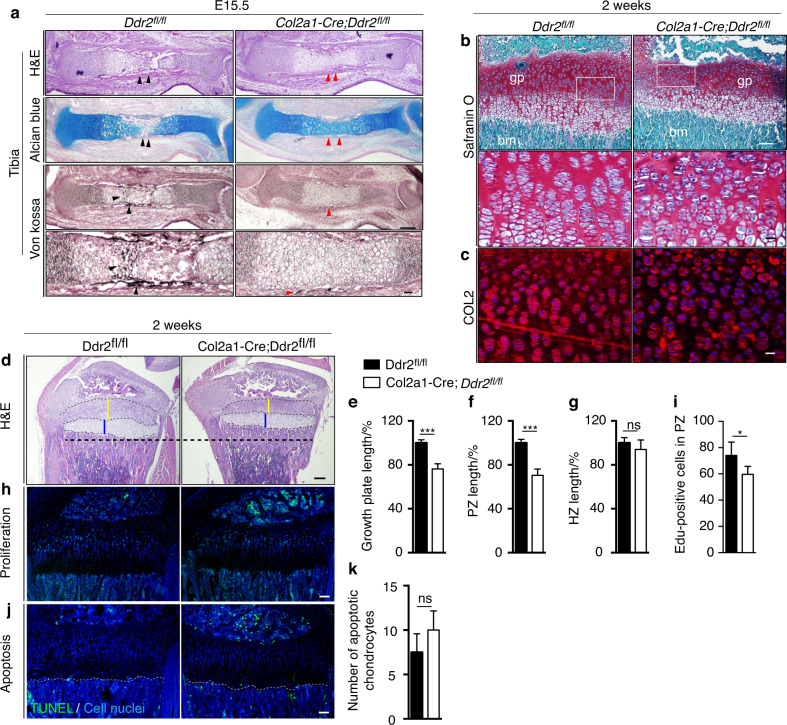
*Ddr2* loss in chondroprogenitor cells results in deficient chondrocyte proliferation, organization, and abnormal type II collagen. **a** Hematoxylin and eosin (H&E), alcian blue and von Kossa staining of developing tibiae at E15.5 showing delays endochondral ossification in developing skeleton. Red arrowheads point to the delay in the formation of primary ossification center (*n* = 3). Scale bar: 200 μm and 100 μm in lower panel. **b** Safranin O staining of tibial growth plate sections of 2-week-old *Col2a1-Cre; Ddr2*^*fl/fl*^ shows chondrocyte disorganization. Boxed region is shown in higher magnification (bottom). Scale bar: 100 μm (top) and 20 μm (bottom). **c** Immunofluorescent staining of tibial growth plate sections of 2-week-old *Col2a1-Cre; Ddr2*^*fl/fl*^ and control littermates with an antibody against type II collagen (red) showing uneven distribution with no immunostaining in between chondrocytes. DAPI (blue) stains cell nuclei. Scale bar: 20 μm. **d**–**g** Hematoxylin and eosin (H&E) staining and quantification showing reduced growth plate lengths in *Col2a1-Cre;Ddr2*^*fl/fl*^ mice at 2 weeks. Scale bar: 200 μm. Unpaired *t*-test ***P < 0.001; ns Not significant. **h** EdU staining (green) of tibial growth plate sections show reduced chondrocyte proliferation in *Col2a1-Cre; Ddr2*^*fl/fl*^ mice. Scale bar: 100 μm. **i** Quantif**i**cation of EdU-positive cells in proliferative zone (*n* = 4). **j** Fluorescent TUNEL staining (green) for detecting chondrocyte apoptosis at chondro-osseous junction (dotted lines). DAPI (blue) stains cell nuclei. Scale bar: 100 μm. **k** Quantification of chondrocyte apoptosis (*n* = 3), unpaired *t*-test, ns, not significant

Localization studies also detected *Ddr2* expression in the resting zone and proliferating chondrocytes. To evaluate the role of *Ddr2* in this cell population, *Col2a1-Cre; Ddr2*^*fl/fl*^ mice were generated. *Col2a1-Cre* activity is first detected in head mesenchyme and notochord at E8.5 with subsequent expansion to cartilage rudiments of all endochondral bones by E12. By E14.5 it is active in all cartilage elements, including resting and proliferating zone chondrocytes. However, it has reduced activity in hypertrophic cells and is inactive in osteoblasts.^38^ *Col2a1-Cre; Ddr2*^*fl/fl*^ mice were developed by breeding *Col2a1-Cre* mice with female *Ddr2*^*fl/fl*^ to generate *Col2a1-Cre; Ddr2*^*fl/+*^ heterozygous mice that were then bred with *Ddr2*^*fl/fl*^ mice. Conditional knockout mice were born at the expected Mendelian frequency. Gross morphology and skeletal phenotypes of 3 month-old *Col2a1-Cre; Ddr2*^*fl/fl*^ mice are shown in Fig. 5 while a more detailed analysis of cartilage during embryonic development and in neonates (P14) is shown in Fig. 6. Analysis of ear cartilage biopsies showed efficient excision of the floxed allele (Supplementary Fig. S6). At 3 months, mutant mice displayed a significant reduction in body length for both males and females (Fig. 5c) and body weight (Fig. 5d). Analysis of trabecular and cortical bone by μCT showed a significant reduction in trabecular BV/TV due to a decrease in trabecular number and thickness and an increase in trabecular spacing, compared with *Ddr2*^*fl/fl*^ controls (Fig. 4e–i). However, no changes in trabecular or cortical BMD were observed (Fig. 5j, l). Trabecular bone changes were similar in magnitude to those observed in tamoxifen-treated *Gli1-Cre*^*ERT*^; *Ddr2*^*fl/fl*^ mice. However, *Col2a1-Cre; Ddr2*^*fl/fl*^ mice exhibited a mildly reduced cortical bone volume (Fig. 5k). We also compared the expression of several bones and cartilage markers and signaling intermediates in RNA extracted from whole tibias (Fig. 5b, m). As expected, *Ddr2* mRNA was reduced by approximately 70 percent in *Col2a1-Cre; Ddr2*^*fl/fl*^ mice. Substantial inhibition was also seen for the osteoblast markers, *Col1a1* and bone sialoprotein (*Ibsp*) and the hypertrophic cartilage markers, *Col10a1* and *Mmp14*. Lastly, *Ddr2* deficiency reduced expression of the Hedgehog pathway signaling intermediates, *Ihh* and *Gli1*.

To further explore the basis for the dwarfism seen in *Col2a1-Cre; Ddr2*^*fl/fl*^ mice, endochondral bone formation was examined during embryonic development and in P14 neonates (Fig. 6). Limb buds from control and *Col2a1-Cre; Ddr2*^*fl/fl*^ embryos were indistinguishable at E13.5 (result not shown), but by E15.5 clear differences were apparent. By this time, a primary ossification center (POC) containing von Kossa-positive mineral was clearly visible in controls (Fig. 6a; black arrowheads). In contrast, initial bone formation was delayed in mutant mice as evidenced by the persistence of alcian blue-stained cartilage and absence of mineral (Fig. 6a; red arrowheads). By P14, this delay in endochondral ossification led to an approximately 24% reduction in growth plate length in *Col2a1-Cre; Ddr2*^*fl/fl*^ mice versus controls (Fig. 6d, e). Linear measurements of individual growth plate histological zones revealed a 30% decrease in the thickness of the proliferative zone in mutant mice (Fig.6f), but no major difference in thickness of the hypertrophic zone (Fig. 6g). To understand the basis for these differences, we assessed chondrocyte proliferation using EdU labeling. For this analysis, mice were intraperitoneally injected with EdU 4 h before sacrifice and cell proliferation was assessed by quantifying EdU-labeled cells in the proliferative zone of growth plates. We found that the number of EdU^+^ cells in mutant growth plates was ~15% lower than controls (Fig. 6h, i), suggesting deficient chondrocyte proliferation. In contrast, chondrocyte apoptosis measured using a TUNEL assay was not significantly different between *Col2a1-Cre; Ddr2*^*fl/fl*^ and control mice (Fig. 6j, k).

The reduced rates of chondrocyte proliferation in *Col2a1-Cre; Ddr2*^*fl/fl*^ mice were accompanied by disruption of chondrocyte columnar organization as seen in higher power images (Fig. 6b, boxed region). These changes were also accompanied by altered distribution of type II collagen as measured by immunofluorescence. In control mice, type II collagen immunostaining was detected around chondrocytes and in the interterritorial matrix. However, in *Col2a1-Cre; Ddr2*^*fl/fl*^ mice, type II collagen was unevenly distributed with strong staining around chondrocytes and diminished staining in the interterritorial matrix (Fig. 6c, red staining). These abnormalities in matrix organization may directly or indirectly inhibit chondrocyte proliferation and organization, both of which may synergistically contribute to overall shortening in mutant growth plates.

### No detectable *Ddr2* function in mature osteoblasts

**Fig. 7:**
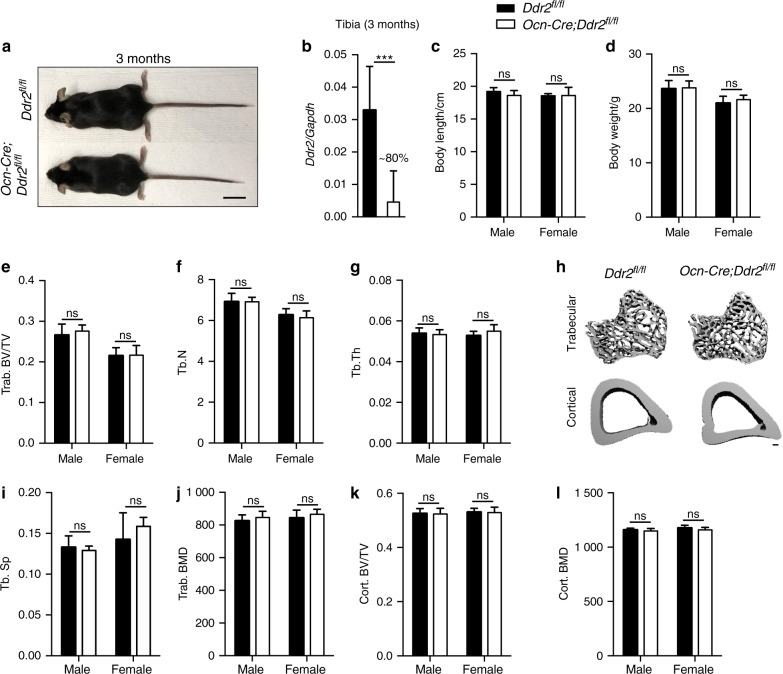
No obvious bone defects with osteoblast specific *Ddr2* knockout. **a** Gross phenotype of 3 month-old *Ddr2*^*fl/fl*^ and *Ocn-Cre;Ddr2*^*fl/fl*^ littermates. *Ocn-Cre;Ddr2*^*fl/fl*^ mice have no skeletal phenotype (*n* = 20, 10 mice/sex). Scale bar, 2 cm. **b** Gene expression analysis of *Ddr2* from whole 3 month-old tibiae to confirm knockout efficiency (*n* = 8 mice). **c**, **d** Measurements of body length (cm) and weight (g) (*n* = 20, 10 mice/sex), unpaired *t*-test. ns Not significant. **e**–**j** Quantitative data of trabecular bone volume fraction (Trab. BV/TV), trabecular number (Tb.N), trabecular thickness (Tb.Th), trabecular spacing (Tb.Sp) and bone mineral density (Trab. BMD), (*n* = 20, 10 mice/sex). **h** 3D micro-computed tomography (microCT) models of the tibial metaphysis and cortical mid-shaft in 3 month-old *Ddr2*^*fl/fl*^ and *Ocn-Cre; Ddr2*^*fl/fl*^ mice. Scale bar 100 µm. **k**, **l** Quantification of cortical bone volume fraction (Cort. BV/TV), and bone mineral density (Cort. BMD). (*n* = 20, 10 mice/sex), unpaired *t*-test, ns Not significant. Data were presented as mean ± SD

As shown above, knockout of *Ddr2* with either *Gli1* or *Col2a1*-*Cre* results in similar phenotypes, including endochondral growth plate-mediated dwarfism and defects in trabecular bone. In both cases, a clear function for *Ddr2* in chondrocyte proliferation is implied. Lineage tracing studies show that *Gli1*-positive progenitors can form chondrocytes (Fig. 3b), so the *Gli1-Cre*^*ERT*^ would be expected to delete *Ddr2* in progenitors as well as chondrocytes^37,39^ while *Col2a1-Cre* would directly inactivate *Ddr2* in chondrocytes. However, the basis for observed bone defects is less clear. On one hand, *Ddr2* could be directly required for osteoblast-mediated bone formation. In fact, we lineage-traced Ddr2-expressing cells to OSX-positive pre-osteoblasts/osteoblasts on select trabecular surfaces (Figs. 1g, 2e) and directly detected Ddr2^+^ cells by IF on trabecular surfaces (Fig. 3c). Also, *Ddr2* is detected in isolated osteoblasts and is necessary for their in vitro differentiation.^30,35^ Alternatively, there is good evidence that at least a fraction of chondrocytes can transdifferentiate into osteoblasts, thereby potentially carrying the recombined *Ddr2* allele into this cell population.^40,41^ To discriminate between these two possibilities, we inactivated *Ddr2* using an osteocalcin promoter-driven Cre (*Ocn-Cre*), which induces efficient recombination in mature osteoblasts and osteocytes, but not skeletal progenitors or chondrocytes.^42^ *Ocn-Cre* induced efficient excision of the floxed *Ddr2* allele as measured in vertebrae-containing tail biopsies from *Ocn-Cre;Ddr2*^*fl/fl*^ mice (Supplementary Fig. S7) and reduced tibial *Ddr2* mRNA by approximately 90 percent (Fig. 7b). Unlike results obtained with *Gli1-Cre*^*ERT*^ or *Col2a1-Cre*, 3 month *Ocn-Cre*;*Ddr2*^*fl/fl*^ knockout mice were indistinguishable from control littermates. There were no differences in body length or weight (Fig. 7a, c, d) or in trabecular or cortical bone parameters (Fig. 7e–l). From these results, we conclude that *Ddr2* is not required for normal bone formation by mature osteoblasts/osteocytes, at least during development and early adult life. Instead, *Ddr2* preferentially functions in *Gli1*-positive skeletal progenitors and chondrocytes.

### Requirement for *Ddr2* in differentiation of isolated chondroprogenitor and skeletal stem cells

**Fig. 8:**
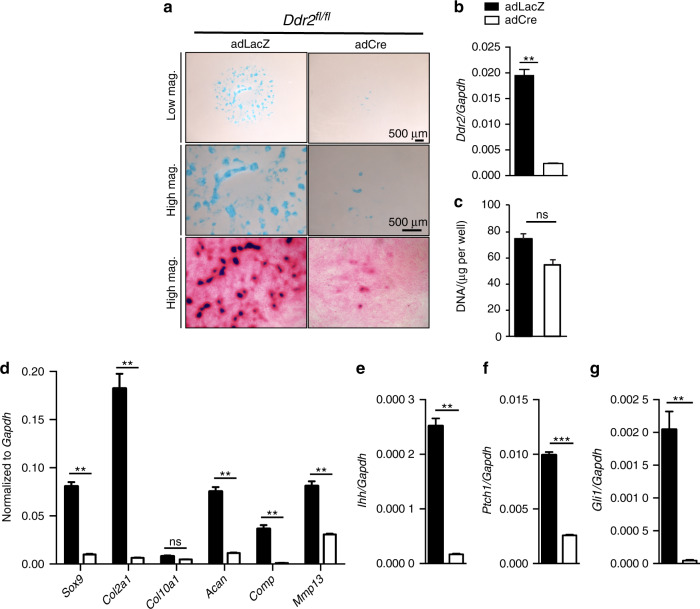
*Ddr2* deficiency inhibits chondrogenic differentiation in micromass cultures. **a** Chondroprogenitor cells isolated from limb buds of E12.5 *Ddr2*^*fl/fl*^ mice were treated with *AdLacz* or *AdCre* and differentiated for 8 days followed by alcian blue staining (top two panels) or were subsequently stained with eosin (bottom panel). Scale bar: 500 μm. **b** Total DNA. **b, c**–**f** Quantitative real-time RT-PCR (qRT-PCR) gene expression analyses of micromass cultures. **b**, **c** Efficient knockdown of *Ddr2*. **c** Gene expression analysis of chondrocyte differentiation markers. **d**–**g** Gene expression analysis shows downregulation of chondrocyte and hedgehog signaling in *Ddr2*-deficient chondrocytes. Unpaired *t*-test *P < 0.05, **P < 0.01; ***P < 0.001; ****P < 0.000 1; ns Not significant

To provide more direct evidence for cell-autonomous functions of *Ddr2* in chondroprogenitors, micromass cultures were established from *Ddr2*^*fl/fl*^ embryo limb buds at E12.5.^43,44^ After transduction with adeno-Cre or adeno-LacZ (control), cells were grown in chondrogenic medium (see Methods). The efficiency of *Ddr2* knockout was confirmed using qRT-PCR (approx. 90 percent decrease in *Ddr2* mRNA, Fig. 8b). Alcian blue staining of micromass cultures showed a dramatic decrease in cartilage nodule formation in adeno-Cre-infected cells (Fig. 8a, top panels). Samples were also stained with eosin to visualize total protein; *Ddr2*-deficient cells were shown to have a diffuse ECM in which cells failed to aggregate (bottom panels). To determine if *Ddr2* inactivation reduced cell number, the DNA content of micromass cultures was measured. However, no significant differences were noted between control and adeno-Cre infected cells (Fig. 8c).

Consistent with *Ddr2* being important for chondrocyte differentiation, expression of the chondrocyte markers, *Sox9*, *Col2a1*, *Aggrecan*, *Comp*, and *Mmp13* were all significantly downregulated (Fig. 8d). Like in vivo results using *Col2a1-Cre; Ddr2*^*fl/fl*^ mice (Fig. 5m), knockout of *Ddr2* in micromass cultures also inhibited expression of the hedgehog signaling intermediates, *Ihh*, *Gli1* and *Ptch1* (Fig. 8e–g).

**Fig. 9:**
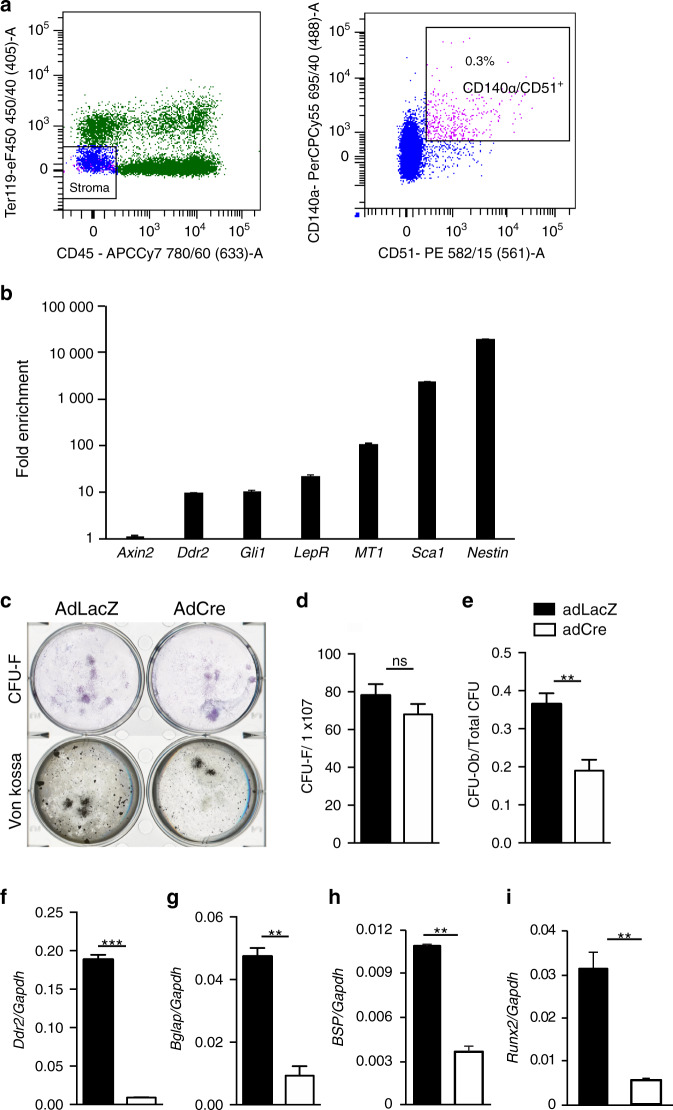
Requirement for *Ddr2* in osteoblast differentiation of purified marrow-derived skeletal progenitors. **a** Flow cytometry analysis and isolation of CD140α/CD51^+^ cells from *Ddr2*^*fl/fl*^ mice. Boxed region (right) shows percentage of BM stem/progenitor cells. **b** Fold enrichment of *Ddr2* and stem cell marker mRNAs in CD140α/CD51^+^ cells. **c**–**e** Colony forming unit-fibroblast (CFU-F) and CFU-osteoblasts (CFU-Ob) assays showing *Ddr2* deficiency inhibits CFU-Ob. **c** Representative images of CFU-F and CFU-Ob. **d** CFU-F. **e** CFU-Ob. **f**–**i** Gene expression analysis of CD140α/CD51^+^ bone marrow cells treated with *AdLacz* or *AdCre* and grown in osteogenic medium. Closed bars, AdLacZ (control); open bars, AdCre. Unpaired *t*-test *P < 0.05, **P < 0.01; ***P < 0.001; ****P < 0.000 1; ns Not significant

*Ddr2*-*LacZ* localization studies identified *Ddr2* expression in the metaphyseal bone marrow, one of the regions known to contain SPCs.^37^ To determine whether *Ddr2* can affect lineage allocation and differentiation of marrow-derived SPCs, we sorted non-hematopoietic (CD45^-^,Ter119^-^), CD140a^+^CD51^+^ cells from the bone marrow of *Ddr2*^*fl/fl*^ mice (Fig. 9a). This cell population has a number of SPC properties; it contains most of the colony forming activity of marrow, has high self-renewal capacity, gives rise to BM stromal cells and bone, supports hematopoiesis and exhibits multipotency (forms osteoblasts, adipocytes and chondrocytes).^45^ Gene expression analysis showed enrichment of *Ddr2* in CD140a^+^CD51^+^ MSCs along with other stem cells markers including *Gli1*, *LepR*, *Sca1* and *Nestin* when compared with total nucleated cells (Fig. 9b). To evaluate the role of *Ddr2* in this population, cells were transduced with adeno-Cre or adeno-LacZ and analyzed for SC properties and multipotency. Adeno-Cre treatment abolished most *Ddr2* expression (95 percent inhibition, Fig. 9f) and reduced lineage allocation of fibroblast colony forming units (CFU-Fs) to osteoblasts (CFU-Ob) without affecting total CFU-Fs (Fig. 9c–e). Similarly, the bone markers, *Bglap*, *Ibsp,* and *Runx*2 were all reduced. These results indicate *Ddr2* is expressed in a skeletal progenitor-enriched cell population and is required for osteoblast differentiation of these cells. They are consistent with in vivo studies showing that *Ddr2* functions in progenitor populations to control the growth and differentiation of bone.

## Discussion

Although it is widely appreciated that the ECM provides important cues for the growth and development of the skeleton, the relative contribution of different collagen receptors to this process is not well-understood. Here we show the fibrillar collagen receptor, DDR2, to be preferentially expressed in resting and proliferating zone chondrocytes and periosteum where it has important cell-autonomous functions necessary for chondrocyte organization and growth as well as subsequent bone formation. Conditional deletion studies revealed a requirement for *Ddr2* in *Gli1*-positive skeletal progenitors and chondrocytes, but no obvious activity in mature osteoblasts. Consistent with a function in progenitor cells, we find that disruption of *Ddr2* in limb bud chondroprogenitors and marrow skeletal progenitors blocks chondrocyte and osteoblast differentiation, respectively. These studies greatly expand our understanding of the cellular functions of this important bone-associated collagen receptor and suggest potential DDR2-mediated strategies for regulating bone formation and regeneration.

Previous studies detected DDR2 in a wide range of mesenchyme-derived tissues including skin, lung, heart, muscle, tooth mesenchyme, and periodontal ligament.^32,46,47^ However, its distribution during development had not been systematically examined. Using a combination of expression studies with *Ddr2*^*LacZ/+*^ mice and lineage tracing, we showed that *Ddr2* is preferentially expressed in resting and proliferating chondrocytes, periosteum and metaphysis. Furthermore, progeny of *Ddr2*-positive cells can form hypertrophic chondrocytes, osteoblasts and osteocytes, consistent with *Ddr2* being expressed in progenitor populations. The earliest *Ddr2* expression was detected at E11.5 and, by E13.5, it was present in all skeletal elements, including growth plate cartilage, metaphyses, and periosteum. This expression pattern persisted into adult life. More detailed analysis showed that, for each bone cell lineage, *Ddr2* was restricted to earlier developmental stages. Thus, growth plate expression was restricted to the resting zone and some proliferating chondrocytes, but low or absent from the hypertrophic zone, while in metaphyseal bone, expression was in the marrow and on the trabecular surface and periosteum of cortical bone, but not in osteocytes. This distribution is markedly different from the collagen-binding integrins, α1β1, α2β1, and α11β1 that are broadly expressed in many tissues. However, the distribution of *Ddr2* may partially overlap with α10β1 integrin, which is preferentially expressed in chondrocytes.^48–50^

Lineage tracing analysis provided further evidence for preferential *Ddr2* expression at early stages in the bone lineage, possibly in SPCs. TAM injection of neonatal *Ddr2*^*mer-iCre-mer*^*;R26R-tdTomato* mice initially labeled cells in the growth plate resting zone, perichondrium, trabecular surfaces, and periosteum in a pattern that was similar to although considerably weaker than that seen when LacZ staining was examined in *Ddr2*^*LacZ/+*^ mice. The more restricted tdTomato staining may be due to low availability of TAM to cartilage or low efficiency of Cre-mediated recombination in *Ddr2*^*mer-iCre-mer*^*;R26R-tdTomato* mice. Nevertheless, at early times after TAM administration, tdTomato immunofluorescence was conspicuously absent from terminally differentiated bone cells including hypertrophic chondrocytes and osteocytes. These cells only became labeled after a 2-month chase period as would be expected if *Ddr2* was expressed in chondrocytes and osteoprogenitors that were precursors to hypertrophic chondrocytes and osteoblasts.

Consistent with localization studies, conditional deletion analysis provided strong evidence for selective activity of *Ddr2* in progenitor populations and chondrocytes. The mice selected for these studies were previously shown to have strong Cre activity in skeletal progenitors (*Gli1-Cre*^*ERT*^), chondrocytes (*Col2a1-Cre*) or mature osteoblasts/osteocytes (*Ocn-Cre*). For *Gli1-Cre*^*ERT*^, lineage tracing previously showed that cells marked with this *Cre* are a major source of skeletal progenitors during embryonic development and in adults.^37^ *Gli1*^+^ cells labeled during embryonic stages give rise to most osteoblasts, chondrocytes, marrow adipocytes and stroma. A separate pool of mesenchymal stem cell-like *Gli1*^+^ cells were identified beneath the growth plate (termed “metaphyseal mesenchymal progenitors”) and shown to be essential for trabecular bone formation in young mice. We compared the distribution of tdTomato in *Gli1-Cre*^*ERT*^;*R26R*-tdTomato mice with *Ddr2*^*mer-iCre-mer*^*;R26R-tdTomato* mice and found considerable overlap. Furthermore, immunofluorescence microscopy showed strong colocalization of Ddr2 and Gli1 in resting and proliferating zone chondrocytes and select metaphyseal cells of the proximal tibia, which is consistent with Ddr2 functioning in Gli1-positive SPCs. The *Col2a1-Cre* we used is expressed early in development beginning at E8.5 and present in all endochondral bone rudiments by E12.5.^38^ Lineage tracing using this *Cre* showed it marks growth plate cartilage and perichondrium by E12.5 and, subsequently, most osteoblasts and marrow stromal cells of endochondral bone, consistent with it being expressed in chondrocytes, some of which can transdifferentiate into osteoblasts.^51^

The phenotypes of TAM-treated *Gli1-Cre*^*ERT*^;*Ddr2*^*fl/fl*^ mice and *Col2a1-Cre; Ddr2*^*fl/fl*^ mice were remarkably similar. *Gli1-Cre*^*ERT*^;*Ddr2*^*fl/fl*^ mice exhibited severe defects in tibial growth plates regardless of whether *Ddr2* was inactivated in embryos (TAM treatment at E12.5-14.5) and analyzed at birth or in neonates (TAM treatment at P1-4) and analyzed at P14. In both cases, growth plate length was reduced from 15-20 percent, comparable to what was seen in globally *Ddr2*-deficient mice. Similarly, growth plate length was reduced by approximately 24% in *Col2a1-Cre; Ddr2*^*fl/fl*^ mice at P14. *Gli1-Cre*^*ERT*^;*Ddr2*^*fl/fl*^ and *Col2a1-Cre; Ddr2*^*fl/fl*^ mice also had similar bone phenotypes when measured at 3 months. In both cases, *Ddr2* knockout reduced trabecular BV/TV by 50%–55% in males and by approximately 40% in females. The only difference noted between *Gli1-Cre*^*ERT*^;*Ddr2*^*fl/fl*^ and *Col2a1-Cre; Ddr2*^*fl/fl*^ mice was a small decrease in cortical BV/TV in the latter group. A similar decrease in cortical bone was also previously observed in globally *Ddr2*-deficient mice.^30^ This difference may be the consequence of reduced efficiency of the *Gli1-Cre*^*ERT*^ or be explained by activity of *Col2a1-Cre* at early embryonic stages versus Gli1-Cre^ERT^, which was not activated by TAM until P1-4. In contrast to these results, *Ocn-Cre; Ddr2*^*fl/fl*^ mice had no detectable bone defects; mice were of normal length and weight and trabecular and cortical bone parameters were indistinguishable from wild type controls.

Further evidence for *Ddr2* having cell-autonomous functions in skeletal progenitor populations came from cell culture studies. *Ddr2* knockout in embryonic limb bud chondroprogenitor cells or PDGFRα^+^/CD51^+^ SPCs purified from marrow prevented chondrocyte or osteoblast differentiation, respectively. In limb bud micromass cultures, *Ddr2* knockout greatly reduced proteoglycan production as measured by Alcian blue staining, prevented cell aggregation and blocked expression of chondrocyte markers and Hedgehog signaling intermediates. Although *Ddr2* deficiency reduced cell proliferation in vivo, results from micromass cultures cannot be explained by a proliferation defect since total cell number as reflect by DNA content was not significantly reduced in with *Ddr2* deficiency. Instead, our results suggest that *Ddr2* is necessary for limb bud cells to aggregate, which is required for subsequent differentiation. Similarly, knockout of *Ddr2* in PDGFRα^+^/CD51^+^ SPCs led to a dramatic decrease in lineage allocation of cell clones to osteoblasts without affecting total CFUs.

One unresolved issue in the above studies is the degree to which *Ddr2* functions in SPCs and is a marker for this cell type versus having more restricted functions in cartilage. While studies with *Ddr2*^LacZ/+^ mice showed expression in resting zone chondrocytes, periosteum and marrow, regions known to contain SPCs, *Ddr2* was not expressed in early mesenchymal condensations during limb development (e.g., compare lack of expression in distal versus proximal limb elements at E13.5, Supplementary Fig. S1). Also, although lineage tracing in *Ddr2*^*mer-iCre-mer*^*;R26R-tdTomato* mice showed initial tdTomato labeling of resting and proliferative zone chondrocytes, periosteum, and metaphysis with subsequent lineage tracing of label into hypertrophic chondrocytes, osteoblasts, and osteocytes, the number of tdTomato cells did not increase over time as would be expected if it were marking an SPC population capable of self-renewal. While it could be argued that the colocalization of Ddr2 with Gli1 and strong skeletal phenotype of tamoxifen-treated *Gli1-Cre*^*ERT*^;*Ddr2*^*fl/fl*^ mice is consistent with *Ddr2* functioning in Gli1-positive SPCs, it cannot be resolved from our studies if *Ddr2* functions in SPCs or in the progeny of these cells, since they are progenitors for chondrocytes and osteoblasts. Lastly, the demonstrated requirement for *Ddr2* in the differentiation of limb bud micromass cultures or CD140a^+^CD51^+^ MSCs both represent results of cell culture assays for SPC properties and do not necessarily mimic in vivo SPC behavior. To resolve whether *Ddr2* actually has a SPC function in vivo will require more detailed analysis, including lineage tracing studies at multiple time points and extensive single cell transcriptome analysis.

The mechanistic basis for the observed defects in cartilage and bone formation associated with *Ddr2* deficiency are not currently understood. From our studies, it is clear that *Ddr2* affects the behavior of skeletal lineage cells at relatively early stages in their lineage, but beyond this little is known. The more detailed examination of growth plate development conducted in *Col2a1-Cre;Ddr2*^*fl/fl*^ mice revealed that initial bony collar formation was delayed in E15.5 embryos. Also, the subsequent reduction in proliferative zone length and dwarfism of *Ddr2*-deficient mice was explained, in part, by an inhibition of chondrocyte proliferation in the absence of changes in apoptosis. This inhibition was accompanied by altered type 2 collagen matrix distribution and abnormal orientation of chondrocytes of resting and proliferating zone chondrocytes. Similar changes in chondrocyte proliferation and matrix organization were previously reported in globally *Ddr2*-deficient mice and in SMED-SL-AC patients, respectively,^27,52^ and may be related to the ability of DDR2 to affect collagen fibril formation either by altering fibrillogenesis or matrix remodeling.^53,54^ Interestingly, knockout of β1 integrins in cartilage also alters chondrocyte organization and matrix deposition, and there is some evidence that DDR2 and β1 integrins functionally interact.^55,56^

Unlike integrins, DDR2 signaling pathways have not been extensively studied. On binding fibrillar collagens, DDR2 is auto phosphorylated with a characteristically slow time course that persists for several hours. This is accompanied by activation of several down-stream events including binding of Src and subsequent activation of the ERK-MAPK pathway.^46^ The importance of MAPK activation is emphasized by the observation that *Ddr2* overexpression can stimulate MAPK-mediated phosphorylation of RUNX2 and PPARγ transcription factors leading to increased osteoblast differentiation and decreased adipogenesis by mesenchymal cells. Consistent with these findings, defects in osteoblast differentiation seen in calvarial cells from *Ddr2*-deficient mice can be rescued by a phosphomimetic RUNX2 mutant (*Runx2*-S301ES319E) that does not require phosphorylation for optimal activity. In contrast, a RUNX2 mutant that cannot be phosphorylated (Runx-S301A,S319A) is not affected by *Ddr2* status.^30^ Earlier studies by our group examined the importance of MAPK signaling in bone formation in vivo using transgenic mice selectively expressing a constitutively-active or dominant-negative form of the MAPK intermediate, MEK1, in bone.^57^ Examination of bone formation in these mice revealed that inhibition of MAPK signaling at E15.5 delayed initial bony collar formation. This result is similar to what we observed in *Col2a1-Cre; Ddr2*^*fl/fl*^ mice where the initial bone formation was also delayed at this time and is consistent with *Ddr2* deficiency being accompanied by a defect in MAPK signaling.

Insight into pathways important for DDR2 signaling may be also gained from gene expression analysis that was conducted on bones from control and *Col2a1-Cre; Ddr2*^*fl/fl*^ mice. In addition to reducing cartilage and bone marker mRNAs, loss of *Ddr2* was associated with reduced expression of Hh pathway signaling intermediates including *Ihh* and *Gli1*. Similar changes in gene expression were noted with *Ddr2* loss in micromass cultures, which suggests it can regulate Hh signaling. In a recent report, cartilage deficiency of the other mammalian DDR homolog, discoidin domain receptor 1, was also associated with defective Hh signaling suggesting that this may be a common pathway regulated by this class of receptors.^58^

## Materials and methods

### Mice

*Ddr2*^*slie/slie*^ mice carrying a spontaneous 150-kb deletion mutation of *Ddr2* exons 1-17 resulting in a null allele was obtained from the Jackson Labs and bred into a C57BL6 background for at least ten generations.^31^ Homozygous (*Ddr2* ^*slie*/*slie*^) mice were obtained from intercrossing heterozygous (*Ddr2*^*slie/+*^) mice. *Ddr2*^LacZ/+^ and *Ddr2*^*fl/fl*^ mice were generated from “knockout-first” ES cell clone *Ddr2*^tm1a(EUCOMM)Wtsi^(EPD0607__B01; European Mutant Mouse Repository) (https://www.eummcr.org). *Ddr2*-*LacZ* knock-in mice were developed by crossing founder mice harboring the Frt- flanked β-galactosidase (*LacZ*)-neo cassette with constitutive *Sox2-Cre* mice while *Ddr2*^*fl/fl*^ mice were generated by crossing founder mice harboring the *loxP*- flanked coding exon 8 (exon 8) with the FLPo delete mice (described in reference^32^). *Ddr2*^*fl/fl*^ mice were backcrossed onto C57BL/6 for at least six generations.

*Ddr2*^*mer-iCre-mer*^ mice harboring MerCreMer cassette knocked in-frame into exon 2 of the *Ddr2* locus were developed using a targeting strategy similar to that reported previously^29^, with changes based on targeting exon 2 of the mouse *Ddr2* gene (see Supplementary Figs. 1 and 2). Briefly, the 4.4 kb left homology arm (LHA) was amplified using primers 5’-AACGCGTTGGATCTGGAGTCTGCAGCCCACCGACAA-3’ and 5’-GGAGATGCAGGCCATGTTGCCCCAACACCTCCCATT-3’. The 4.0 kb right homology arm (RHA) was amplified using primers 5’-ACTCCACAGTGCCAAAGATGGTGCCTGAAGCCATGA-3’ and 5’-GCGGCCGGGTTCCCAAGATCTCAGGTAAGCTTTTGT-3’. The arms were cloned individually into pCR-BluntII-TOPO (Life Technologies), selecting the desired orientation by restriction enzyme screening. The MerCreMer-neo^R^ cassette was excised from the vector, pBKSII-MerCreMer-Mcl-neo (kindly provided by Dr. Sylvia Evans, UCSD), by NotI and partial NcoI (cutting only at sequence position 60) digestion. This NcoI/NotI MerCreMer-neo^R^ cassette was ligated into the MluI/NotI sites of the pCR-BluntII-LHA construct, using an MluI-HinDIII-NcoI linker adapter (annealed 5’-CGCGTAAGCTTCCAC-3’ and 5’-CATGGTGGAAGCTTA-3’). The resulting construct was digested with NotI, dephosphorylated, and ligated to the EagI insert of pCR-BluntII-RHA to produce pCR-BluntII-LHA-MerCreMer-neo^R^-RHA; the correct orientation was selected by restriction enzyme screening. To provide negative selection, a diphtheria toxin A coding cassette (PspOMI/NotI fragment, kindly provided by Dr. Ju Chen, UCSD) was then ligated into the dephosphorylated NotI site of pCR-BluntII-LHA-MerCreMer-neo^R^-RHA, ensuring that the preserved NotI site was distal to the RHA. The resulting targeting vector was linearized with NotI, electroporated into 129R1 mouse embryonic stem cells, and individual G418-resistant clones selected. Clones were screened initially by PCR, and the positive clones were verified by Southern blot, using KpnI digestion with probe C and BamHI digestion with probe D (Supplementary Fig. 2a). One targeted clone was microinjected into C57Bl/6 blastocysts, and these blastocysts were implanted into pseudopregnant female hosts. Chimeric pups were bred and agouti pups screened for the targeted mutation. The neomycin resistance (neo^R^) cassette was removed by breeding with a FLPe deleter mouse (kindly provided by Dr. Ju Chen, UCSD). The resulting mouse was backcrossed into C57Bl/6 for at least ten generations. The Transgenic Core and Embryonic Stem Cell shared resource at UCSD assisted in the production of this knockin mouse. Validation of MerCreMer protein expression in cardiac fibroblasts from a tamoxifen-treated a positive *Ddr2*^*mer-iCre-mer*^ mouse is shown in Supplementary Fig. 2b*Rosa26-CAG-loxP-stop-loxP-tdTomato* (Ai14: R26R-tdTomato) mice, a gift from Dr. Noriaki Ono (U. Michigan), have been described previously.^59^ To obtain *Ddr2*^*mer-iCre-mer*^; *R26R*^*tdTomato*^ reporter mice, we crossed *Ddr2*^*mer-iCre-mer*^ heterozygous mice with the Ai14:*R26R*^*tdTomato*^ line.

*Ddr2* tissue-specific conditional knockouts were generated by crossing mice carrying the *Ddr2* floxed allele with the following Cre driver mouse lines: *Gli1-Cre*^*ERT*^ (gift from Dr. Yuji Mishina, U. Michigan), *Col2a1-Cre* (gift from Dr. Ernestina Schipani, U Michigan) *and Ocn-Cre* (gift from Dr. Kurt Hankenson (U. Michigan).^38,42,60^ Most mice used in our experiments were analyzed on C57BL/J6 background, except *Col2a1-Cre*; *Ddr2*^*fl/fl*^ mice were analyzed on mixed background. All mice were housed under a 12 h light cycle in compliance with the Guidelines for the Care and Use of Animals for Scientific Research. All protocols for mouse experiments were approved by the Institutional Animal Care and Use Committee of the University of Michigan.

### Genotyping

Production and genotyping of *Col2a1-Cre*, *Gli1-Cre*^*ERT*^, *Ocn-Cre* and Ai14: R26R-tdTomato mice have been described previously.^38,42,59,60^ Genomic DNA was prepared from tail snips or ear punches using DNA REDExtract-N-Amp™ Tissue PCR Kit (Sigma-Aldrich, St. Louis, MO) according to the manufacturer’s instructions. Tissue samples were incubated in extraction buffer for 10–30 min at room temperature (RT), heated at 95 °C for 3 min followed by neutralization. Polymerase chain reaction (PCR) analysis was then used to genotype mice. Information about primers used for genotyping *Ddr2*^*fl/f*^, *Ddr2*^LacZ/+^ and *Ddr2*^*mer-iCre-mer*^ mice is presented in Supplementary Materials, Table 1. The genotyping of *Ddr2*^*slie/slie*^ mice was performed using qRT-PCR using conditions defined by Jackson Labs.

### Tamoxifen administration

Tamoxifen-free base (Sigma T5648) was dissolved in absolute ethanol. The tamoxifen solution was mixed with corn oil (Sigma C8267) and vortexed thoroughly to prepare a stock solution of 40 mg·mL^−1^. For administration of tamoxifen by oral gavage, 20 mg·mL^−1^ progesterone (Sigma P3972) was added to the tamoxifen stock solution. An appropriate volume of tamoxifen-progesterone solution was administrated by oral gavage to pregnant females for 3 days beginning from E12.5 and pups were harvested at birth. For newborn mice, four daily tamoxifen injections were administrated intragastrically using a 29 G needle (BD insulin syringe). The dosing conditions used were previously described.^61^

### Micro-computed tomography analysis

A total of 20 tibiae collected from 3 month-old male and female mice (*n* = 10/gender) were fixed in 10% formalin at 4 °C overnight. Tibiae were scanned using a Scanco Model 100 (Scanco Medical) to evaluate bone parameters of proximal trabecular bone and mid-shaft cortical bone. Scan settings were as follows: voxel size of 12 μm, 70 kVp, and integration time of 500 ms. For the trabecular bone of proximal tibia, a contouring tool was used to trace the trabecular bone in 30 CT slices below the growth plate to evaluate bone volume fraction (Trab. BV/TV; Bone volume/ Total volume), trabecular number (Tb.N), trabecular thickness (Tb.Th), trabecular spacing (Tb.Sp), and trabecular bone mineral density (Trab. BMD). The analysis of the cortical bone included 30 CT slices at the tibial mid-shaft to assess bone volume fraction (Cort. BV/TV) and cortical bone mineral density (Cort. BMD). The mean BMD value is in units of mg HA per ccm. A threshold of 180 for tibial trabecular and 280 for cortical bone analysis was used throughout the whole study. The mouse genotype was not specified during μCT analysis to avoid examiner bias.

### LacZ (β-galactosidase) staining and detection

LacZ staining of heterozygous *Ddr2-LacZ* (*Ddr2*^*LacZ/+*^) mice was performed as previously described.^62^ Samples were fixed in 2% paraformaldehyde and 0.2% glutaraldehyde in 0.1 mol·L^−1^ phosphate buffer pH 7.4 at 4 °C. For LacZ staining, samples were incubated in a freshly prepared X-gal solution containing X-gal substrate (UltraPure, X-Gal, Invitrogen) for overnight at 37 °C. For cryostat sections, samples were decalcified with 20% ethylenediaminetetracetic acid (EDTA) (pH 7.2) from 3 days to 2 weeks according to the mouse age, embedded in optimal cutting temperature compound (Tissue-Tek), cryosectioned at 10–12 μm thickness, subjected to LacZ staining and counterstained with Vector Nuclear Fast Red.

### Histology and immunostaining

Long bones were fixed in 4% paraformaldehyde (PFA) for 48 h at 4 °C. Specimens were decalcified in 10% EDTA (pH 7.2) (Fisher, S316-212) and were then processed for paraffin embedding. Specimens were sectioned at 5 μm using a microtome, de-paraffinized and hydrated in ethanol series and in distilled water. For histological analysis, the sections were stained with hematoxylin and eosin (H&E) according to the standard procedures. For Safranin O staining, sections were stained with Safranin O (0.1% Safranin O) for 20 min and Fast Green (0.02% Fast Green FCF) for 5 min. For von Kossa staining, sections were stained with 5% silver nitrate solution for 1 h under strong light, rinsed with distilled water, and stained with 5% sodium thiosulfate solution for 5 min, and counterstained with nuclear fast red staining for 5 min. For immunofluorescence, sections were subjected to heat-induced antigen retrieval using citrate buffer (target retrieval solution, Dako) following the manufacturer instructions, washed with PBS and then incubated with blocking buffer containing 2%–5% normal donkey serum, 1% bovine serum albumin (BSA), 0.01% tween in 1 BSP for 1 h at RT in a humid box. After blocking, the sections were then incubated at 4 °C overnight using Anti-COL2 (1:100, ab34712, Abcam), Anti-DDR2 (1:200 LS B15752, LSBio) and Anti-Gli1 (1:100, Santa Cruz SC-515751). The sections were incubated with species-matched secondary antibody (Invitrogen) for 1 h at RT. The slides were mounted using ProLong™ Gold Antifade Mountant with DAPI (Life technologies) for cell nuclei staining. For colocalization studies in *Ddr2*^*mer-iCre-mer*^; *R26R*^*tdTomato*^ reporter mice, frozen sections were prepared as described for LacZ studies, stained with anti-OSX antibody and approriate secondary antibodies before analysis by immunofluorescence microscopy using a Nikon 50i microscope.

### Proliferation and apoptosis assay

For proliferation, mice were injected intraperitoneally with 5-ethynyl-2’-deoxyuridine (component A, Invitrogen, # C10337) and sacrificed 4 h after injection. EdU-labeled cells were detected using Click-iT® EdU Alexa Fluor® 488 Imaging Kit (Invitrogen, # C10337) according to the manufacture instruction. For apoptosis, Click-iT® Plus TUNEL assay was used on tissue sections following the manufacture instructions (Invitrogen, # C10618). The sections were mounted with DAPI (Life technologies).

### Fluorescence-based ELF97 TRAP for osteoclasts

Tissue sections were stained with tartrate-resistant acid phosphatase (TRAP) using an ELF97 endogenous phosphatase detection kit (Invitrogen) combined with reagents from an acid phosphatase, Leukocyte (TRAP) Kit (Sigma-Aldrich). Briefly, ELF97 endogenous phosphatase substrate was added to a mixture of 11 mmol·L^−1^ sodium nitrate, 112 mmol·L^−1^ sodium acetate, and 76 mmol·L^−1^ sodium tartrate solutions in ddH_2_O. Sections were incubated with the TRAP mixture in a dark humid chamber for 15 min at RT, washed with PBS, mounted with aqueous medium.

### Micromass culture for chondrogenesis

Micromass culture was performed according a published protocol.^44^ Primary chondroprogenitors were isolated from E12-limb buds of *Ddr2*^*fllfl*^ embryos. Briefly, limb buds were harvested under sterile conditions and digested in 1 U·mL^−1^ dispase II-containing solution (dispase II enzyme from Gibco) for 40 min at 37 °C. The cells in the digestion solution were spun down, resuspended in DMEM F12 media plus 10% FBS (growth medium), and filtered using a 40 μ cell strainer. The cell density and volume was adjusted to have 2 × 10^7^ cells per mL. The cells were transfected with Ad5-CMV-LacZ (AdLacZ) (control) or Ad5-CMV-CRE (AdCre) adenoviruses at MOI of 100. The adenoviruses were prepared at a titer of 1 × 10^11^ pfu per mL and obtained from the Vector core, University of Michigan. Cells were grown for 3 day in growth medium, transferred to differentiation medium (growth medium supplemented with 1 mmol·L^−1^ β-glycerol phosphate and 0.25 mmol·L^−1^ ascorbate) and grown for an additional 8 day before analysis. For alcian blue staining, the cultured cells were fixed with 3.7% formalin in BPS, washed with BPS and 0.2 mol·L^−1^ HCl for 5 min and stained with alcian blue staining as described previously.^44^

### Flow cytometry/FACS analysis

Mouse tibiae were flushed using a 21 G needle to collect BM cells into digestion buffer containing 2 mg·mL^−1^ collagenase IV/ 3 mg·mL^−1^ dispase in 1X PBS. Multiple cycles of digestion with agitation were performed at 37 °C (7 min each) to obtain single cell suspension. Single cell suspensions were transferred into DMEM medium containing 10% calf serum and maintained at 4 °C until the rest of the tissue was completely digested. Red blood cells were lysed in 1:1 NH_4_Cl hypotonic solution. Cells were filtered into clean 50 mL conical tubes, centrifuged at 1 100 r·min^−1^, 4 °C, for 10 min, resuspended in staining buffer (2 mmol·L^−1^ EDTA, 0.5% BSA in 1X PBS), and transferred to FACS tubes for staining. The following cell-surface markers were used: For exclusion hematopoietic cells: TER119-AF700 (Biolegend, Clone:TER119, 1:100) and CD45-APC-Cy7 (Biolegend, Clone:30-F11, 1:200). For identifying MSCs: CD140a-Biostin (Biolegend, Clone:APA5, 1:200) and CD51-PE (Biolegend, clone:RMV-7, 1:200). MSCs (CD45-Ter119-CD31-PDGFRα^+^ CD51^+^) were used for colony and gene expression assays.

### Colony forming unit (CFU) assays

CFU assays were performed with briefly cultured MSCs from bone marrow (CD45-Ter119-CD31-PDGFRα^+^ CD51^+^). Cells were seeded at a density of 1 × 10^3^ cells per well in 6 well plates and maintained in DMEM/10% FBS medium at 37 °C with 5% CO_2_. The medium was changed every 2–3 days. After 14 days, cells were stained with Giemsa Stain Solution (Sigma #R03055) to detect CFU-Fibroblasts. For CFU-Ob detection, cells were grown in 50 μg·mL^−1^ ascorbic acid and 10 mmol·L^−1^ β-glycerophosphate for 14 days. Cultures were incubated at room temperature in 2% Alizarin Red S (pH 4.2 with 10% ammonium hydroxide) for 0.5 h.

### Gene expression analysis

Total RNA was extracted using Trizol (Invitrogen) according to the manufacturer’s protocol. Total RNA (1 μg) was used to make complementary DNA (cDNA) using TaqMan™ reverse transcription reagents (Applied Biosystems). mRNA levels were determined using quantitative real-time PCR (qRT-PCR) with TaqMan probes on an ABI 7500 thermocycler (Applied BioSystems). *Gapdh*, the housekeeping gene, was used to normalize the expression of levels of target genes.

### Statistical analysis

All data were analyzed using GraphPad Prism software (version 6.Oe, La Jolla, CA, USA), and results were reported as mean ± SD. A samples size of 20 (*n* = 20, *n* = 10/gender) was used for microCT analysis and at least three mice were used for histological analysis. Two-tailed unpaired *t*-test was used to analyze the difference between the controls and mutant groups. A probability was considered statistically significant when *P < 0.05; **P < 0.01, ***P < 0.001, ****P < 0.000 1. ns. not significant. Results are presented as mean ± SD or SEM as indicated.

## Supplementary information

Supplementary Materials Mohamed et al
