# Supplementary material for: The collagen receptor, discoidin domain receptor 2, functions in Gli1-positive skeletal progenitors and chondrocytes to control bone development
Source: Bone Res. 2022 Feb 9;10:11. doi: 10.1038/s41413-021-00182-w (PMC8828874; doi:10.1038/s41413-021-00182-w)
Supplement: Supplementary file 1 — Supplementary Materials Mohamed et al [file 41413_2021_182_MOESM1_ESM.pdf]

Supplementary Figure S1

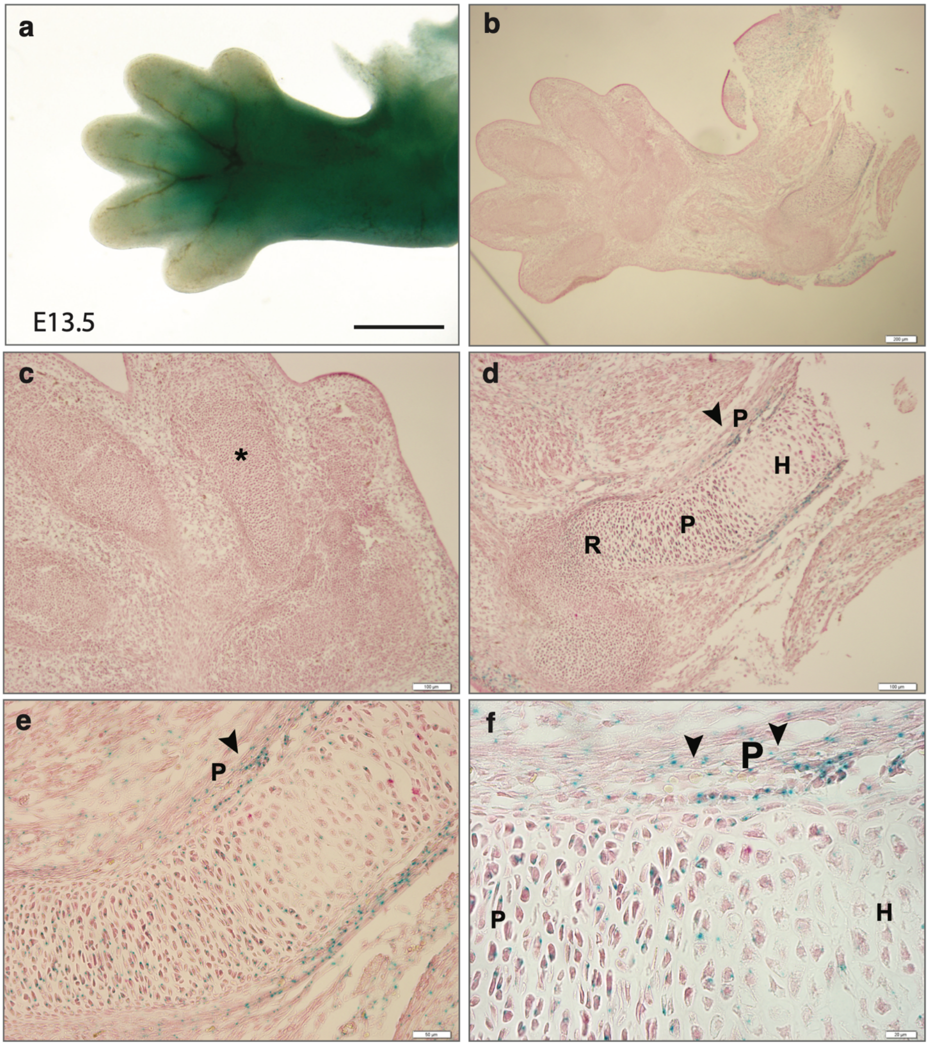

Supplemental Figure S1. Fetal *Ddr2-LacZ* skeletal expression. **a**, Whole-mount X-gal staining of *Ddr2*$^{Lacz/+}$ forelimb at E13.5. Scale bar: 500µm. **b-f**, X-gal staining of cryostat section of humerus from P13.5 embryo. **c,** No detectable staining in mesenchymal condensations of developing digits (asterisk). **d-f**, LacZ staining reveals *Ddr2* expression in resting (R) and proliferative (P) zones and perichondrium (P) (black arrowheads), but no or low staining in the hypertrophic (H) zone of cartilage template. Scale bar: 200µm in **a,** 100µm in **c** and **d**, 50µm in **e,** and 20µm in **f**.

Supplementary Figure S2

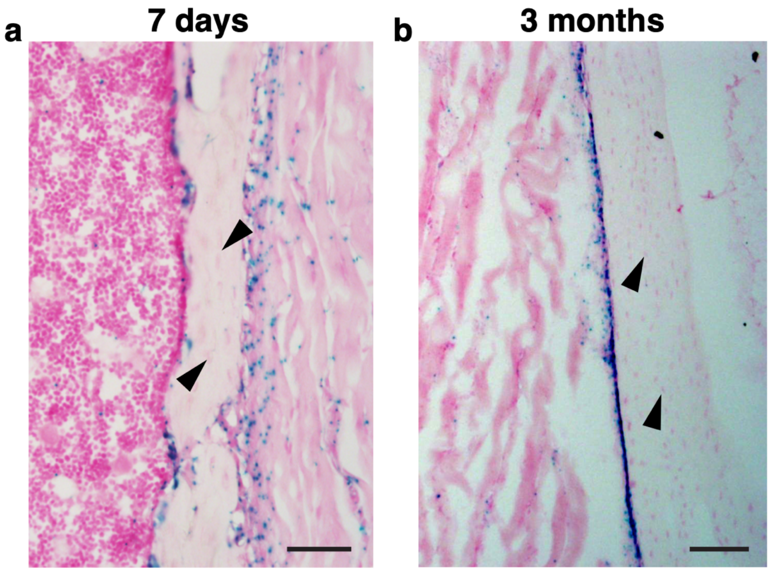

Supplemental Figure S2. Osteocytes have undetectable *Ddr2* expression. a,b, X-gal staining of cryostat sections of long bone from 7 day-old (**a**) and 3month-old (**b**) *Ddr2*$^{Lacz/+}$ mice. To improve substrate penetration, X-gal staining was performed after sectioning. Scale bar**:** 50µm. Black arrowheads point to osteocytes in the cortical bone.

Supplementary Figure S3

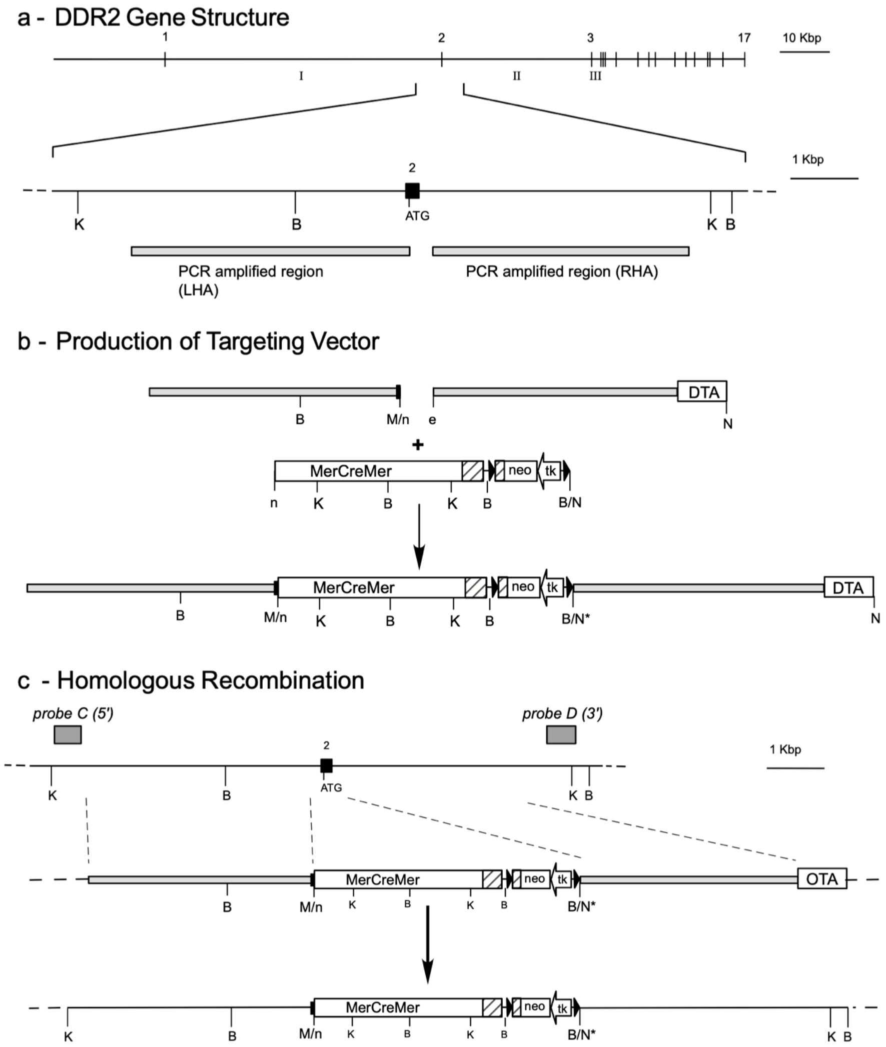

Supplemental Figure S3: ***Ddr2-Cre^{mer-iCre-mer}*** **construction- Strategy for knock-in of MerCreMer into DDR2 exon 2.** Black box, exon; Hatched box, poly(A) site; Black triangle, Frt site; K, KpnI; B, BamHI; M, MluI; n, NcoI; e, EagI; N, NotI; N*, destroyed NotI; neo, G418 resistance; tk, viral TK promoter; DTA, diphtheria toxin A; LHA, left homology arm; RHA, right homology arm.

Supplementary Figure S4

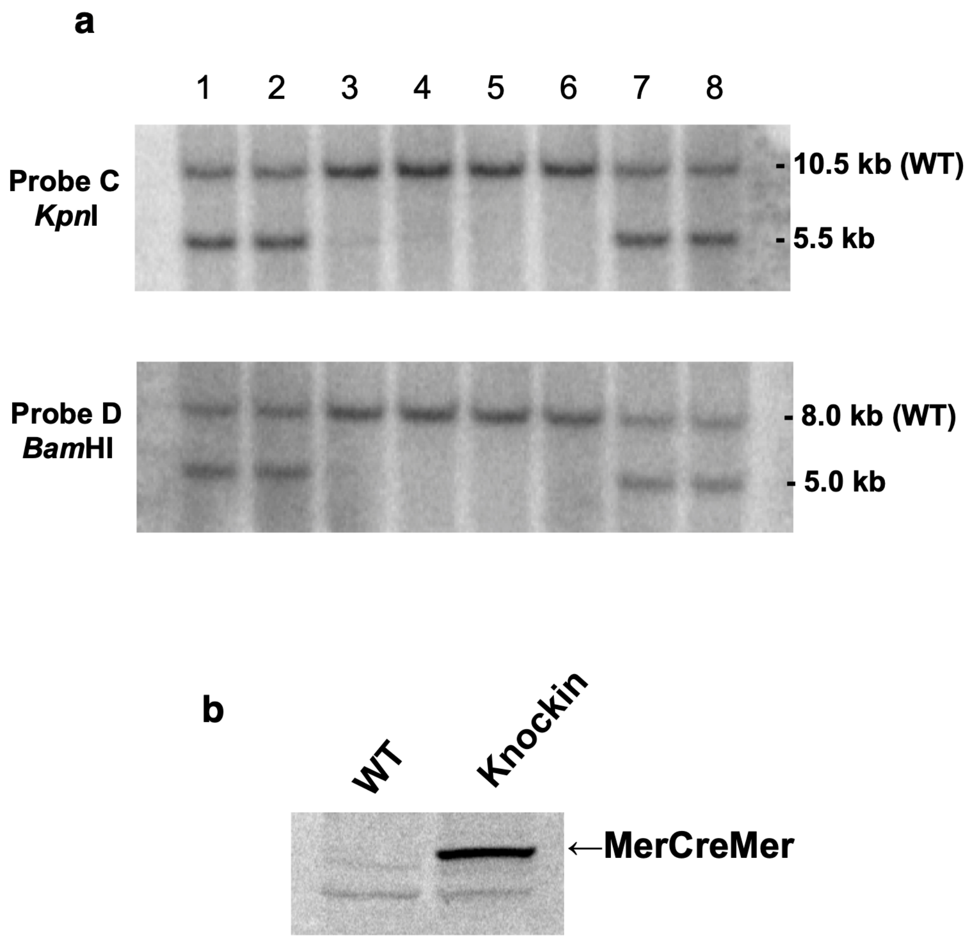

Supplemental Figure S4: ***Ddr2-Cre***$^{mer\text{-}iCre\text{-}mer}$ **characterization. a**, Southern blot verification of potential ES clones identified initially via PCR. Probes indicated on the left are those shown in Supplementary Figure S1C, with the indicated restriction digestion of genomic DNA. Clones 1, 2, 7, and 8 are targeted (positive). Clones 3-6 are wildtype (WT, negative). **b**, Verification of MerCreMer protein expression in cultured cardiac fibroblasts from a positive mouse (Knockin). The primary antibody in part B directed to the mutated estrogen receptor was obtained from Santa Cruz Biotechnology (ERα, C-211, cat. no. sc-787).

Supplementary Figure S5

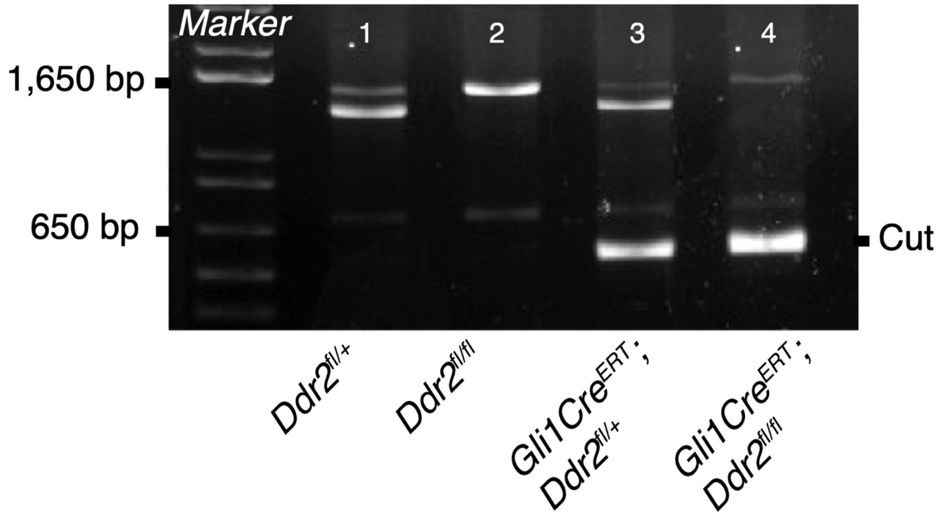

Supplemental Figure S5. PCR analysis of *Gli1-Cre^{ERT};Ddr2$^{fl/fl}$* mice. Genotyping PCR for *Ddr2$^{fl/fl}$* and *Gli-Cre^{ERT}* alleles showing the Cre-mediated excision of *Ddr2* exon 8. *Ddr2$^{fl/fl}$* mice were bred with *Gli1Cre^{ERT}* mice and treated with tamoxifen at P1–P4. DNA was extracted from ear cartilage at 3 months. The following PCR products were observed; 1600 bp *Ddr2* floxed allele (upper band), wild type *Ddr2* allele (1500 bp, lower band) and recombined Ddr2 floxed allele generated in presence of *Gli1CreERT* and tamoxifen (600 bp, bottom band).

Supplementary Figure S6

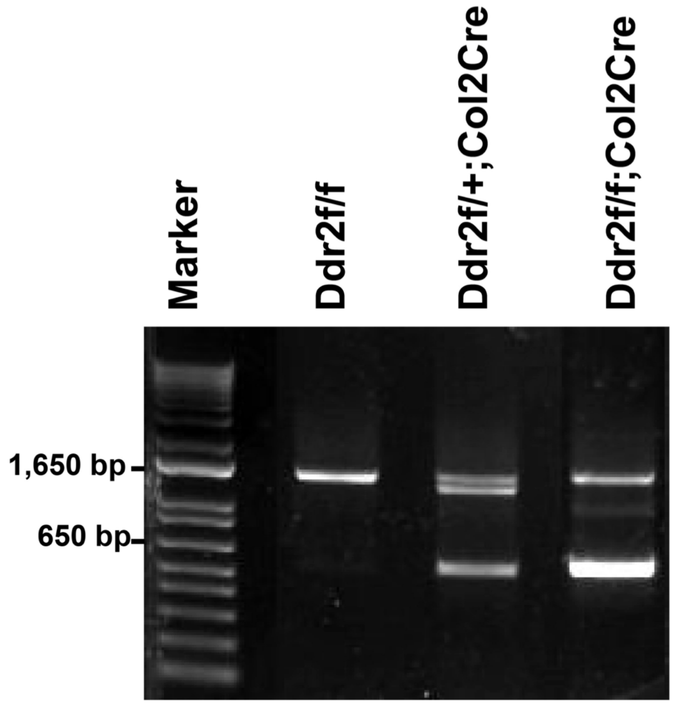

Supplementary Figure S6. ***Col2a1-Cre;Ddr2**$^{fl/fl}$* **PCR of recombination.** Same analysis as if Fig S7 except using *Col2a1-Cre* to induce recombination.

Supplementary Figure S7

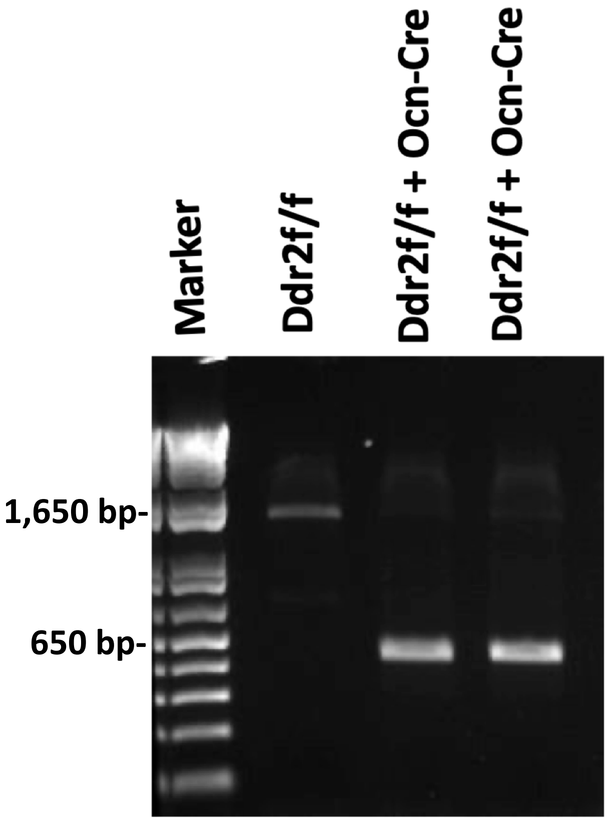

Supplementary Figure S7. ***OC-Cre;Ddr2$^{fl/fl}$*** **PCR of recombination.** Same analysis as if Fig S6,7 except that tail biopsies were the source for DNA and Ocn-Cre was used to induce recombination.

Supplemental Table 1: Primers used for genotyping

Ddr2 flox	
Primers	Oligonucleotide sequence **5`→ 3`**
ARM1F	TCCCGCTGAAAGGTCATGAG
ARM1R	TTGTTTTCAAATACCACAGCAAGA

Ddr2-Lacz	
Primers	Oligonucleotide sequence **5`→ 3`**
LacZ P1	GACGACTCCTGGAGCCCGTCAGTA
LacZ P2	TTGTTTTCAAATACCACAGCAAGA

Ddr2-mer-iCre-mer	
Primers	Oligonucleotide sequence **5`→ 3`**
Forward	CCCCCTGAACCTGAAACATAA
Reverse	GGCCAAAGAAACCCTAGATGA
